# Features Constituting Actionable COVID-19 Dashboards: Descriptive Assessment and Expert Appraisal of 158 Public Web-Based COVID-19 Dashboards

**DOI:** 10.2196/25682

**Published:** 2021-02-24

**Authors:** Damir Ivanković, Erica Barbazza, Véronique Bos, Óscar Brito Fernandes, Kendall Jamieson Gilmore, Tessa Jansen, Pinar Kara, Nicolas Larrain, Shan Lu, Bernardo Meza-Torres, Joko Mulyanto, Mircha Poldrugovac, Alexandru Rotar, Sophie Wang, Claire Willmington, Yuanhang Yang, Zhamin Yelgezekova, Sara Allin, Niek Klazinga, Dionne Kringos

**Affiliations:** 1 Department of Public and Occupational Health Amsterdam UMC Amsterdam Public Health Research Institute, University of Amsterdam Amsterdam Netherlands; 2 Department of Health Economics Corvinus University of Budapest Budapest Hungary; 3 Laboratorio Management e Sanità Institute of Management and Department EMbeDS Scuola Superiore Sant'Anna Pisa Italy; 4 Danish Center for Clinical Health Services Research Department of Clinical Medicine Aalborg University Aalborg Denmark; 5 Department of Psychiatry Aalborg University Hospital Aalborg Denmark; 6 OptiMedis AG Hamburg Germany; 7 Hamburg Center for Health Economics University of Hamburg Hamburg Germany; 8 School of Medicine and Health Management Tongji Medical College Huazhong University of Science and Technology Wuhan China; 9 Department of Clinical and Experimental Medicine University of Surrey Surrey United Kingdom; 10 Nuffield Department of Primary Care and Health Services University of Oxford Oxford United Kingdom; 11 Department of Public Health and Community Medicine Faculty of Medicine Universitas Jenderal Soedirman Purwokerto Indonesia; 12 Independent Researcher Minneapolis, MN United States; 13 Institute of Health Policy, Management and Evaluation University of Toronto Toronto, ON Canada

**Keywords:** COVID-19, pandemic, internet, performance measures, public reporting of health care data, public health, surveillance, health information management, dashboard, accessibility, online tool, communication, feature, expert

## Abstract

**Background:**

Since the outbreak of COVID-19, the development of dashboards as dynamic, visual tools for communicating COVID-19 data has surged worldwide. Dashboards can inform decision-making and support behavior change. To do so, they must be actionable. The features that constitute an actionable dashboard in the context of the COVID-19 pandemic have not been rigorously assessed.

**Objective:**

The aim of this study is to explore the characteristics of public web-based COVID-19 dashboards by assessing their purpose and users (“why”), content and data (“what”), and analyses and displays (“how” they communicate COVID-19 data), and ultimately to appraise the common features of highly actionable dashboards.

**Methods:**

We conducted a descriptive assessment and scoring using nominal group technique with an international panel of experts (n=17) on a global sample of COVID-19 dashboards in July 2020. The sequence of steps included multimethod sampling of dashboards; development and piloting of an assessment tool; data extraction and an initial round of actionability scoring; a workshop based on a preliminary analysis of the results; and reconsideration of actionability scores followed by joint determination of common features of highly actionable dashboards. We used descriptive statistics and thematic analysis to explore the findings by research question.

**Results:**

A total of 158 dashboards from 53 countries were assessed. Dashboards were predominately developed by government authorities (100/158, 63.0%) and were national (93/158, 58.9%) in scope. We found that only 20 of the 158 dashboards (12.7%) stated both their primary purpose and intended audience. Nearly all dashboards reported epidemiological indicators (155/158, 98.1%), followed by health system management indicators (85/158, 53.8%), whereas indicators on social and economic impact and behavioral insights were the least reported (7/158, 4.4% and 2/158, 1.3%, respectively). Approximately a quarter of the dashboards (39/158, 24.7%) did not report their data sources. The dashboards predominately reported time trends and disaggregated data by two geographic levels and by age and sex. The dashboards used an average of 2.2 types of displays (SD 0.86); these were mostly graphs and maps, followed by tables. To support data interpretation, color-coding was common (93/158, 89.4%), although only one-fifth of the dashboards (31/158, 19.6%) included text explaining the quality and meaning of the data. In total, 20/158 dashboards (12.7%) were appraised as highly actionable, and seven common features were identified between them. Actionable COVID-19 dashboards (1) know their audience and information needs; (2) manage the type, volume, and flow of displayed information; (3) report data sources and methods clearly; (4) link time trends to policy decisions; (5) provide data that are “close to home”; (6) break down the population into relevant subgroups; and (7) use storytelling and visual cues.

**Conclusions:**

COVID-19 dashboards are diverse in the why, what, and how by which they communicate insights on the pandemic and support data-driven decision-making. To leverage their full potential, dashboard developers should consider adopting the seven actionability features identified.

## Introduction

Since the outbreak of COVID-19, public reporting of pandemic-related indicators such as new cases, death counts, and testing rates has surged. This heightened level of activity attests to the core function of governments to protect the public’s health and safety as well as their critical role of providing information to achieve this end [1-4]. The uses and advantages of publicly reporting health information are known. They include enabling international comparisons [5,6]; monitoring and improving the quality of care [1,6,7]; fostering accountability and transparency [8-10]; empowering the public to form an opinion on and build trust in their government’s response; and supporting individuals to make informed, risk-minimizing behavior changes [11,12].

Dashboards are a dynamic modality for reporting data visually; they are typically designed as a single screen with the aim of quickly and effectively presenting users with critical information to act upon [13-15]. Unlike static reporting modalities, such as articles or reports, dashboards have the potential to present real-time (or near–real-time) data updates at a glance [15]. In the health sector, dashboards have been relied on for health system performance assessments [15,16], internal management [17,18], and responses to earlier outbreaks [19,20].

In 2020, the urgent worldwide need for COVID-19 data, coupled with the penetration of the internet [21], digitalization of health information systems [22,23], and access to open-source web-based software [24], has enabled unmatched speed, scale, and diversification of actors in the development of dashboards to monitor and report on the COVID-19 pandemic. As a result, public web-based dashboards have been widely adopted as a reporting modality for COVID-19 data. Examples extend well beyond national, regional, and local governments to include dashboards by international organizations (eg, the World Health Organization (WHO) [25]), academia (eg, the Johns Hopkins Coronavirus Resource Center [26,27]), and industry (eg, Deloitte [28]), as well as independent initiatives (eg, nCoV2019.live [29]).

Although COVID-19 dashboards may be widely accessible, their effective *use* to modify the course of the pandemic through the translation of data to information, information to opinions, and opinions to decision-making is determined by their actionability. To be actionable, the information should be both *fit for purpose*—meeting a specific information need—and *fit for use—*placing the right information into the right hands at the right time and in a manner that can be understood [30-32]. In other words, the mere accessibility of COVID-19 dashboards does not guarantee data-informed decision-making [12,33]. Although communication sciences, health promotion, and the emerging field of health care performance intelligence offer insights into the effective delivery of information [14,33-36], the factors that make dashboards actionable in the context of COVID-19 have yet to be rigorously assessed.

In this study, we set out to explore the state of the art of publicly available web-based COVID-19 dashboards and identify the features conducive to their actionability. To do so, we took a “snapshot” of this dynamic landscape and assessed COVID-19 dashboards in July 2020. The resulting overview of the dashboard landscape served both to take stock of their use in this initial period and to accelerate their progress in the phases still to come. With these aims, the study was guided by four key questions: (1) Why and for whom were COVID-19 dashboards developed? (2) What information do they provide? (3) How is this information analyzed and presented? and (4) What are the common features of highly actionable dashboards?

## Methods

### Study Design

We conducted an observational descriptive assessment and scoring using nominal group technique (NGT) [37,38] on a global sample of COVID-19 dashboards. Each dashboard was reviewed using a study-specific assessment tool that was piloted and validated among a panel of scorers (n=17) prior to its use [37,38]. NGT was chosen over other consensus methods (eg, Delphi) for scorers to independently appraise a subset of dashboards using the assessment tool and collectively discuss what makes them actionable through a series of workshops [38,39]. All workshops were conducted virtually rather than face-to-face in accordance with pandemic-related public health measures.

#### Panel of Scorers

A panel of scorers was assembled through an existing international network of health care performance intelligence researchers [40]. The scorers had common expertise and training in health care performance data and the use of these data for management and governance. Collectively, the scorers (8 women, 9 men) were of 15 nationalities and were proficient in more than 20 languages (Bosnian, Catalan, Chinese, Croatian, Danish, Dutch, English, French, German, Indonesian, Italian, Kazakh, Malay, Montenegrin, Norwegian, Portuguese, Romanian, Russian, Serbian, Slovenian, Spanish, Swedish, and Turkish). This enabled the dashboards to be assessed in their original languages rather than through translations, avoiding the use of translation software and its limitations when used with data visualizations.

#### Inclusion and Exclusion Criteria

We defined a COVID-19 dashboard based on the following criteria: (1) reporting of key performance indicators related to the COVID-19 pandemic; (2) the use of some form of data visualization; (3) dynamic reporting, meaning the data are updated regularly; and (4) public availability in a web-based format. No restrictions were placed on a dashboard’s primary level (eg, international, national, regional, or local) or the type of organization responsible for its development (eg, international, governmental, academia, news or media, industry, or private initiative). We excluded dashboards that were available only via mobile apps (eg, Telegram) or that required users to log in (eg, Facebook). Dashboards beyond the language competencies of the panel of scorers were also excluded.

### Step One: Dashboard Sampling

Our search strategy for dashboards aimed to be thorough but not exhaustive. This was in line with our aim of exploring the state of the art of public web-based COVID-19 dashboards. An initial list of dashboards was collected through sampling conducted from May 19 to June 30, 2020. Three methods were applied: (1) surveying the authors; (2) surveying other international networks of public health, health services, and system researchers and practitioners (Young Forum Gastein, European Public Health Association, and European Network of Medical Residents in Public Health); and (3) snowballing of sources identified through (1) and (2). The sampling survey was developed using a Google Forms data collection tool and disseminated by email (Multimedia Appendix 1).

The consolidated list of dashboards was screened by one team member with the aims to confirm the inclusion criteria were met; exclude duplicates; and assess the available languages for each dashboard against the panel’s competencies. Dashboards were labeled as red (exclude), green (include), or yellow (obtain second opinion). A second team member assessed dashboards labeled yellow, from which a final joint decision on inclusion or exclusion was made.

### Step Two: Developing an Assessment Tool

An assessment tool was developed by drawing primarily on two existing theoretical models. From communication sciences, we applied the Lasswell model (1948) [41], which states that for mass communication processes to be understood, each element of “who (says) what (to) whom (in) which channel (with) what effect” has to be presented and understood. These five elements—the communicator, message, medium, audience, and effect—informed the basis of the assessment tool’s considerations. We tailored these considerations to the communication of COVID-19 data by drawing on the emerging discipline of performance intelligence in health [36,42]. Specifically, we incorporated key considerations from a definition of actionability and its notions of fitness for purpose and use (Barbazza et al, unpublished data, 2021). The resulting considerations are in line with existing health information instruments (eg, [43,44]), although they were tailored to the aims of the study.

These considerations were clustered to depict COVID-19 dashboards by their general characteristics and a description of why, what, and how data is communicated, followed by an appraisal of their overall actionability (Table 1). Actionability scores were defined on a Likert scale from “not actionable” (score=1) to “extremely actionable” (score=5) and assigned based on the scorer’s judgement of the considerations assessed and their expert opinion of the dashboard’s fitness for purpose and use. Scores were accompanied by a written statement explaining the rationale behind the response. In line with the study’s aim to consolidate key features of highly actionable dashboards, the scoring was merely a means to this end: the panel’s individual appraisal of actionability facilitated the clustering of the actionability of the dashboards as low (score=1 or 2) or high (score=4 or 5) for further collective deliberation on their common features.

**Table 1:** Overview of the assessment tool.

Cluster	Considerations
General characteristics	Level (scale) of focus Responsible organization and type Languages available Scope of web page information
Why	Purpose of use of the dashboard Intended audience (user)
What	Indicator titles Data sources Availability of metadata Frequency of data updates
How	Use of time trend for analysis Geographic level (scale) of analysis Types of possible breakdowns Use of visualizations Degree of interactivity Use of simplicity techniques
Actionability score	Overall appraisal of actionability

An Excel-based tool (Microsoft Corporation) was developed to record our findings. Each consideration of the assessment tool was formulated as a question with defined answer options. The tool included the underlying theory for the considerations by referring back to the concepts applied and available evidence [1,2,5,16,30,31,33,45-55] (Multimedia Appendix 2) to remind the panel of the significance of each consideration and aid the assessment and scoring process.

### Step Three: Piloting and Calibrating

A prototype of the assessment tool was piloted by two authors on five dashboards. The extracted data were reviewed jointly with two other team members. This resulted in refinements to the phrasing of the questions and answer options. A second iteration of the assessment tool was then piloted with the panel of scorers on a sample of 18 dashboards representing a range of contexts, levels, and organization types. Each dashboard was independently reviewed by two scorers. Prior to piloting, a virtual training session with the panel of scorers was organized, recorded, and disseminated to serve as a resource. Each scorer was given six days (June 17-22, 2020) to review their two assigned pilot dashboards.

The pilot data were reviewed to assess the consistency of responses (ie, scorers of the same dashboard recorded equivalent answers) and meaningfulness of the answers (ie, the answer categories were meaningfully differentiated between dashboards). Where possible, the open-ended answer options of the tool were further specified into categorical values based on recurrent themes in the pilot data set. Definitions were added for key terms based on comments by the scorers. The reviewed pilots and tool amendments were returned to the panel of scorers, and a follow-up meeting was organized to discuss the reviews.

### Step Four: Data Extraction and Round One Scoring

Each scorer was assigned between 5 and 12 dashboards to assess. The dashboards were distributed with first order priority given to the language competencies of each scorer. To synchronize the assessment, the scorers were given a 2-week period to complete data extraction. The assessment was limited to each dashboard’s main page, and a “one-click-away policy” was applied by which content accessible within one click of the main page was also assessed. To store a record of the dashboard on the date it was reviewed, the main page of each dashboard was archived, generating a permanent and publicly available record of its contents [56].

### Step Five: Preliminary Analysis and First Consensus Workshop

The data records from each scorer were consolidated by the lead authors into a master data set for analysis and subsequently underwent a series of data quality checks to detect data entry errors, inconsistences, or missed fields. In all instances where errors were detected, corrections were suggested and discussed jointly; once agreed upon, the changes were entered into the master data set.

The findings were totaled and averaged by research question. Free text fields and comments were analyzed in a deductive and inductive approach: topics explored in the tool (Multimedia Appendix 2) were used to guide the deductive thematic analysis [57], and new themes that emerged were identified using an inductive approach [58]. This included an analysis of indicator titles using an existing classification of types of pandemic-related information [3]. Due to the observed variability in phrasing of indicator titles and calculations, key performance indicators were grouped by themes.

A workshop with the panel of scorers was organized to discuss the preliminary results and distribution of actionability scores. During the workshop, panelists individually shared the rationale for their scoring of dashboards with low (score=1 or 2) and high (score=4 or 5) actionability. The common features of dashboards scored as highly actionable were discussed to further calibrate the panel’s scoring of the actionability. From this discussion, a working list of actionability features was consolidated.

### Step Six: Round Two Scoring and Second Consensus Workshop

All panelists were returned their original data records and given 1 week to revisit their initial actionability scoring, drawing on the discussion during the workshop. Panelists were given the opportunity to increase each score, lower it, or leave it the same. Following rescoring, the distributions of the scores were recalculated. The data records for the top dashboards (score=5) following this second round were consolidated and provided to the panel, together with the working set of actionability features. A second consensus workshop was convened and, in a similar way to the previous workshop, a round table was conducted for each scorer to share their views. This was followed by a joint discussion to reach agreement on the common features of highly actionable dashboards.

## Results

### Identified Dashboards

**Figure 1:**
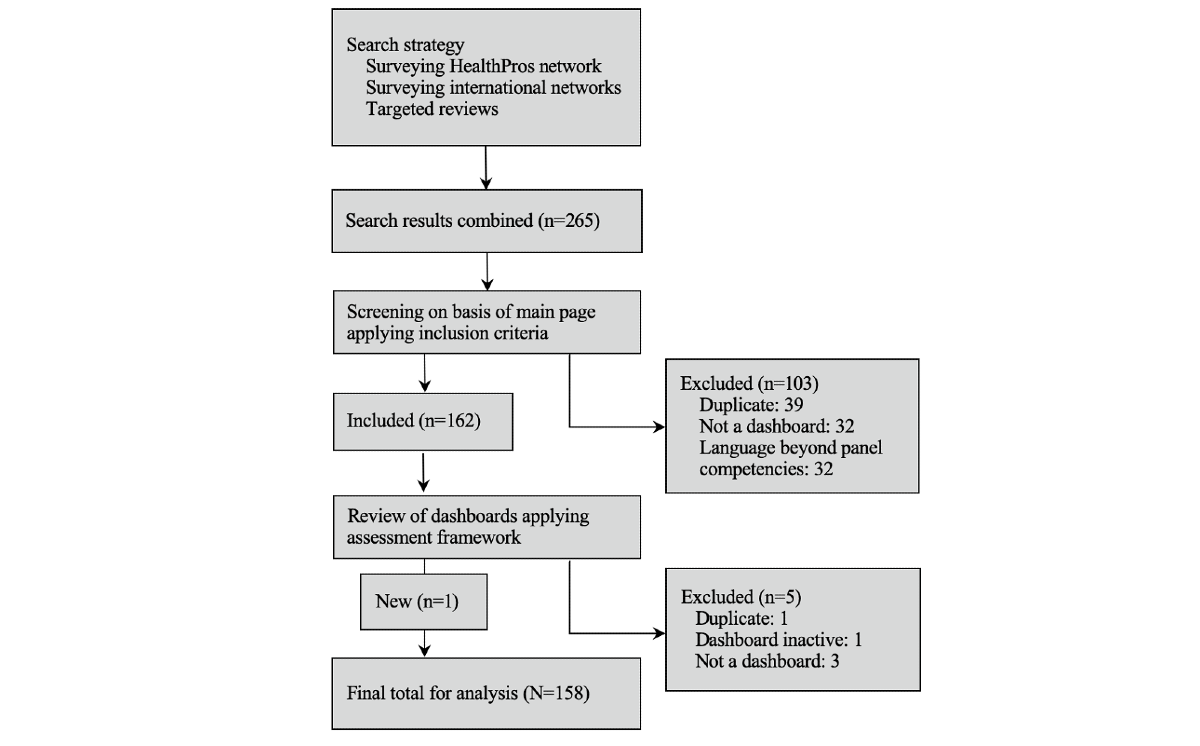
Flow diagram of COVID-19 dashboard sampling.

Using our multimethod search strategy, we initially identified 265 COVID-19 dashboards. More than 40 respondents contributed to the sampling survey, including all members of the study team and international public health experts. Following screening of each dashboard’s main page, 103 dashboards were excluded. The remaining 162 dashboards were distributed among the panel of scorers for full review. During the assessment process, 5 additional dashboards were excluded and 1 new dashboard was included. A final total of 158 dashboards was included for further analysis (Figure 1).

Data extraction and the first round of scoring were conducted in a 2.5-week period between July 6 and 23, 2020. The data extract and archived version of each dashboard were referred to throughout the study. Therefore, any updates following this date were not accounted for. The 158 dashboards were assessed in 22 different languages, predominately in English (n=85, 53.8%), followed by Russian (n=11, 7.0%), Spanish (n=9, 5.7%), French (n=9, 5.7%), and Chinese (n=6, 3.8%). A full listing of the dashboards assessed is available in Multimedia Appendix 3.

### General Description of the Assessed COVID-19 Dashboards

Table 2 summarizes key characteristics of the 158 dashboards assessed. Our sample included dashboards reporting on 53 countries in all 6 WHO regions [59]. On the date of the review, the severity of the pandemic with regard to total cases and deaths varied widely between location as reported in Multimedia Appendix 3.

More than half of the dashboards (93/158, 58.9%) were developed for use at the national level. Nearly two-thirds of the dashboards (100/158, 63.3%) were developed by government authorities at the national, regional, or municipal level. New initiatives or organizations formed in response to COVID-19 accounted for 10.1% (16/158) of the dashboards assessed [29,60-74].

With regard to language, only one-fifth of the dashboards were available in more than one language with full functionality (32/158, 20.3%). In terms of their scope of information, gauged according to the content of the dashboard as well as information to which users were redirected through affiliate links, almost all the dashboards were epidemiological in focus (156/158, 98.7%), followed by providing information on infection control measures and health system management (65/158, 41.1%, and 49/158, 31.0%, respectively).

**Table 2:** Characteristics of the assessed COVID-19 dashboards (N=158) from 53 countries.

Characteristic		Value, n (%)
**Region^a^**		
	Global	20 (12.7)
	Europe and Central Asia	63 (39.9)
	North and South America	45 (28.5)
	Western Pacific	22 (13.9)
	Southeast Asia	4 (2.5)
	Africa	3 (1.9)
	Eastern Mediterranean	1 (0.6)
**Level**		
	International	25 (15.8)
	National	93 (58.9)
	Regional (provincial, state, county)	33 (20.9)
	Municipal (city, district)	7 (4.4)
**Type of organization**		
	International organization	7 (4.4)
	Governmental	100 (63.3)
	Academia	9 (5.7)
	News or media outlet	14 (8.9)
	Industry	9 (5.7)
	Independent initiative	16 (10.1)
	Other	3 (1.9)
**Languages available with full functionality^b^**		
	One language	126 (79.7)
	Two languages	22 (13.9)
	Three or more languages	10 (6.3)
**Additional languages available with reduced functionality^c^**		
	One or more languages	16 (10.1)
**Scope of information^d^**		
	Epidemiological information	156 (98.7)
	Infection control measures	65 (41.1)
	Health system management	49 (31.0)
	Social and economic implications	31 (19.6)
	Population behavioral insights	25 (15.8)
	Other	28 (17.7)

^a^Country status and region according to the WHO classification [59].

^b^Full functionality: the webpage is equivalent in the different languages.

^c^Reduced functionality: the webpage is available in additional languages but with less information and fewer functionalities compared to the main languages.

^d^According to the WHO classification [3].

### Uses and Users of COVID-19 Dashboards

A quarter of the dashboards (45/158, 28.5%) explicitly stated the intended purpose of their reporting. Of these 45 dashboards, the statements spanned three main themes: (1) high-level reporting to create trust and aid overall compliance (25/45, 56%); (2) sub-national reporting targeting policy interventions, including benchmarking (12/45, 27%); and (3) individual-risk assessment (8/45, 18%).

Only 14.6% (23/158) of the dashboards explicitly stated the intended audience (end users). Target users predominately included the general public (20/23, 87%) and, in a few instances, more specific audiences such as travelers or subject matter experts (6/23, 26%). When examined by the level of reporting, national-level dashboards were less likely to explicitly state the intended audience (9/93, 10%), while international- and municipal-level dashboards were more likely to do so (7/25, 28%, and 2/7, 29%, respectively).

Of the 158 dashboards assessed, 20 (12.7%) reported both the purpose and intended user explicitly. The profiles of these dashboards, in terms of their levels of reporting and the types of organizations that developed them, did not differ from the characteristics of the general sample. For the remainder of the analysis, the sample of dashboards was aggregated rather than subdivided by the intended purpose of the use and audience, due to the limited explicit statements of both.

### Content and Data of COVID-19 Dashboards

#### Key Performance Indicators

Table 3 summarizes the frequency of indicator themes reported by the dashboards. See Multimedia Appendix 4 for illustrative examples of indicator titles. On average, the dashboards reported on 5.3 indicator themes (maximum 15, minimum 1). Almost all the dashboards reported public health and epidemiological indicators (155/158, 98.1%), particularly those that reported on cases and deaths. These account for the only high-frequency indicator themes (indicators present in more than two-thirds of the assessed dashboards). Medium-frequency indicator themes (themes reported in more than one-third but less than two-thirds of dashboards) were related to hospital care (hospitalizations, admissions to infection control units), testing (total tests, testing rates), and spread and death (recovered and active cases).

Only 4.4% of the dashboards (7/158) reported indicators related to social and economic impacts. Indicator themes included employment and hardship relief (eg, [28,75]) and transport, trade, and international travel (eg, [28,75]). Indicators of behavioral insights were also infrequently reported (8/158, 5.1%). Indicator themes included two main types: (1) self-reported adherence related to restrictions (eg, [76,77]) or health and well-being status (eg, [75]) and (2) observed public adherence to restrictions assessed through mobility data or reported breaches of restrictions (eg, [60,78]).

Some use of composite scores to signal overall risk levels or the current status by sector (eg, health, economy) was identified, although this use was infrequent (eg, [28,61,79]).

**Table 3:** Frequency of indicator themes reported for the 158 dashboards assessed.

Information type and cluster		Indicator themes		Value, n (%)		Frequency^a^	
**Public health and epidemiological**							
	Spread and death		Cases (all confirmed cases)		150 (94.9)		High
			Deaths		136 (86.1)		High
			Recovered (healed, cured)		91 (57.6)		Medium
			Active cases		56 (35.4)		Medium
			Mortality rate (case fatality rate)		24 (15.2)		Low
			Reproduction rates (attack rate)		12 (7.6)		Low
			Future projections/risk models		5 (3.2)		Low
			Doubling rate		3 (2.0)		Low
	Testing		Testing (total number tested, PCR^b^ tests)		80 (50.6)		Medium
			Testing rates (positivity, negative tests)		43 (27.2)		Medium
			Tests–pending results		17 (10.8)		Low
			COVID-19 antibody tests (serology tests)		1 (0.6)		Low
	Risk management		Self-quarantine (isolation notices)		18 (11.4)		Low
			Contact tracing		6 (3.8)		Low
**Health system management**							
	Hospital care		Hospitalized (admissions, discharges)		74 (46.8)		Medium
			Admitted to ICU^c^ (critical condition)		47 (29.7)		Medium
			On a ventilator		14 (8.8)		Low
	Health system capacity		Hospital bed capacity (availability)		12 (7.6)		Low
			ICU bed capacity		10 (6.3)		Low
			Ventilator capacity (available ventilators)		5 (3.2)		Low
			Non–COVID-19 service usage		4 (2.5)		Low
			Personal protective equipment stock		2 (1.3)		Low
			Testing stock		2 (1.3)		Low
**Social and economic impact**							
	N/A^d^		Employment and hardship relief		7 (4.4)		Low
			Transport, trade, and international travel		3 (1.9)		Low
**Behavioral insights**							
	N/A		Observed public adherence to restrictions		4 (2.5)		Low
			Self-reported adherence to restrictions		2 (1.3)		Low
			Self-reported health and well-being status		2 (1.3)		Low

^a^Low: ≤33%; medium: 34%-66%; high: ≥67%.

^b^PCR: polymerase chain reaction.

^c^ICU: intensive care unit.

^d^N/A: not applicable.

#### Data Sources and Metadata

One quarter of the dashboards did not explicitly report the source of their data (39/158, 24.7%). National-, regional-, and municipal-level government-run dashboards predominately reported the use of data sourced from official public health authorities. International dashboards predominately reported the use of data sourced from the WHO [25] or the Johns Hopkins Centre for Systems Science and Engineering [26].

Less than half of the dashboards (63/158, 39.9%) specified metadata (data dictionaries, indicator specifications) in the format of notes, footnotes, or linked additional web pages to provide further information on the methodology by which an indicator was calculated. Of the 158 dashboards, 39 (24.7%) did not report their data sources or metadata details. The majority of dashboards updated their data daily and explicitly stated the update frequency and time of the last update.

### Types of Analysis and Presentation of Data on COVID-19 Dashboards

Table 4 summarizes the types of analysis and presentation of data. The dashboards predominately reported indicators over time (138/158, 87.4%), and most of these breakdowns were by day (128/138, 92.8%). Of the dashboards, 40% reported data on two geographic levels (eg, national and regional or regional and municipal). In the case of national-level dashboards (n=93), geographic breakdowns predominately included regional comparisons (73/93, 79%), with some municipal-level (28/93, 30%) and international-level (25/93, 27%) comparisons. Breakdowns by neighborhood (post–code-level) were reported in only a few instances (4/93, 4%).

In addition to geographic breakdowns, more than half of the dashboards (96/158, 60.8%) analyzed data by other breakdowns: on average, three types of breakdowns were included. Of these 96 dashboards, the most common breakdowns included by age (79/96, 82%), sex (71/96, 74%), and mode of transmission (26/96, 27%). Other breakdowns, although less frequently reported, included race, ethnicity, long-term care facilities, health care workers, comorbidities, and socioeconomic status.

As per our inclusion criteria, all dashboards used some form of visualization. On average, two types of visualizations were included per dashboard. These included graphs or charts (134/158, 84.8%), maps (111/158, 70.3%), and tables (95/158, 60.1%). Almost half of the dashboards (76/158, 48.1%) did not include written descriptions to clarify either the quality or meaning of the data, while 31/158 dashboards (19.6%) provided both.

More than half of the dashboards (104/158, 65.8%) used some technique to simplify the data. In these 104 dashboards, color-coding was most often used (n=93, 89.4%), followed by size variation (n=40, 38.5%). The majority of dashboards (126/158, 79.7%) included some element of user interaction. These elements mostly included the possibility to present more information (eg, pop-up windows), change the information (eg, different breakdowns), or change the display (eg, switch from table to map).

**Table 4:** Summary of analysis and presentation of dashboard information.

Considerations		Value, n (%)
**Time trend analysis availability (N=158)**		
	Time trend analysis available	138 (87.3)
	No time trend analysis	20 (12.7)
**Use of time trend analysis (n=138)^a,b^**		
	By day	128 (92.8)
	By week	33 (23.9)
	By month	19 (13.8)
**Geographic levels (scales) of analysis (N=158)^b^**		
	International (multicountry)	54 (34.2)
	National	118 (74.7)
	Regional	117 (74.1)
	Municipal	54 (34.2)
	Neighborhood	13 (8.2)
	Other	5 (3.2)
**Number of levels (scales) of analysis per dashboard (N=158)**		
	1 level	34 (21.5)
	2 levels	65 (41.1)
	3 or more levels	59 (37.3)
**Disaggregation availability per dashboard (N=158)**		
	1 or 2 types of disaggregation	48 (30.4)
	3 or 4 types of disaggregation	42 (26.6)
	5 or more types of disaggregation	6 (3.8)
	No disaggregation options	62 (39.2)
**Disaggregation options (n=96)^a,b^**		
	Age	79 (82.3)
	Sex	71 (74.0)
	Mode of transmission	26 (27.1)
	Long-term care facilities	16 (16.7)
	Ethnicity	12 (12.5)
	Race	10 (10.4)
	Health workers	9 (9.4)
	Comorbidities	9 (9.4)
	Socio-economic status	2 (2.1)
	Other	23 (24.0)
**Visualization features (N=158)^b^**		
	Graphs/charts	134 (84.8)
	Maps	111 (70.3)
	Tables	95 (60.1)
	Video/animations	10 (6.3)
**Use of narratives to interpret data (N=158)**		
	Yes, to clarify the quality of the data only	28 (17.7)
	Yes, to clarify the meaning of the data only	23 (14.6)
	Yes, to clarify both the quality and the meaning	31 (19.6)
	No	76 (48.1)
**Simplification techniques used (n=104)^a,b^**		
	Use of color-coding	93 (89.4)
	Size variation	40 (38.5)
	Icons	6 (5.8)
**Interactive options (n=126)^a,b^**		
	More information	115 (91.3)
	Change of information	61 (48.4)
	Change of display	44 (34.9)

^a^Subset of applicable dashboards (ie, 138 dashboards that *do* use time trends).

^b^Percentages for these considerations do not total to 100%, as multiple considerations could be present per dashboard.

### Features of Actionable Dashboards

In the first round of scoring, 21 of the 158 dashboards assessed (13.3%) were scored with the highest actionability score (score=5), and 18 dashboards (11.4%) received the lowest score (score=1), for a mean score of 3.01 (SD 1.20). The second round of scoring resulted in a final total of 20 dashboards that were scored as the most actionable. A quarter of the dashboards (40/158, 25.3%) were scored differently: 24 scored lower, and 16 scored higher. All 17 panelists completed both rounds of scoring. Details on the distribution of scoring by panelist and between rounds are summarized in Multimedia Appendix 5.

The panel workshop following the first round of scoring resulted in a total of 18 features that characterized highly actionable dashboards. After rescoring, these features were further discussed among the panel to consolidate the list in terms of their description and importance as well as its consistency and completeness as a set. A final total of seven key features common to highly actionable dashboards were agreed upon (Table 5). There was consensus among the panelists that some dashboards excelled in certain features over others. These dashboards are noted as illustrative examples.

**Table 5:** Seven features of highly actionable COVID-19 dashboards.

Number	Feature	Explanation	Examples
1	Know the audience and their information needs	Dashboards with a known audience and explicit aim had focus and continuity in their content, analysis and delivery. Techniques such as guiding key questions or overall composite scores clearly communicated the decision they intended to support. Multilanguage functionality and exact timing of updating signaled an awareness and intent to encourage their regular use by the intended decision maker.	#HowsMyFlattening [60], Covid Act Now [61], State of California [79].
2	Manage the type, volume, and flow of information	The selection of a concise number of indicators brought focus and importance to the information and the possibility to view indicators together at a glance. The use of indicators in moderation, although still spanning varied types of information, was especially effective. The ordering of information, from general to specific or in sections based on theme, made the flow of information intuitive.	Covid Act Now [61] reports on five key indicators. Deloitte [28] and the City of Vancouver [78] included a range of types of information.
3	Make data sources and methods clear	A clear source of data and explanation of an indicator’s construction, including potential limitations, was found to be an important component of trust in the dashboard and clarity in its reporting. This information can be provided in short narratives that support users to understand what is in fact being presented.	Denmark [80], France [76], Spain [81], and media pages of the Canadian Broadcasting Corporation [82] and the New York Times [83] paid attention to narrating the calculation of indicators.
4	Link time trends to policy (decisions)	Reporting data over time together with the introduction of key infection control measures facilitated an understanding of their effect (or lack thereof). This was found to be conducive to generating public support for infection control measures.	ABC News [84] and Sledilnik [62] embed policy measures over time. The City of Toronto [85] reports city targets.
5	Provide data “close to home”	To inform individuals of risks in their immediate surroundings, granular geographic breakdowns are needed. Data that are highly aggregated are difficult to understand. Maps (over tables and charts) were most effective to provide geographic information.	The United Kingdom [86] offers post–code-level breakdowns. Germany [87] provided city- and borough-level information for Berlin.
6	Break down the population to relevant subgroups	Providing data with the possibility to explore varied population characteristics made indicators relatable to individual users. It enables understanding of risks and trends based on one’s own demographics. It can also facilitate equity-driven decision-making by exposing differences among the population.	Ethnicity and race breakdowns were provided in New Zealand [75] and various US dashboards [79,88-92]. #HowsMyFlattening [60] provided breakdowns on economic status.
7	Use storytelling and visual cues	A concise narrative explaining the significance of a trend supports users to understand the importance of the information. Bare statistics without a narrated analysis leave the burden of interpretation solely to the user. Brief explanations on the meaning of trends used in combination with visual techniques, such as intuitive color schemes and icons, supported ease of interpretation.	Covid Act Now [61] narrates the significance of trends. The State of Colorado [88] uses colored icons to signal the direction of trends.

## Discussion

### Principal Findings

With this study, we set out to assess the state of the art of public web-based COVID-19 dashboards globally during the initial stage of the pandemic (July 2020) and identify features common to the dashboards that were found to be highly actionable. We assessed 158 dashboards, each operating in a different context. Their differences aside, the dashboards analyzed in this study ultimately share a common aim: to serve as both a communication tool and call for individual and collective action to respond to the COVID-19 pandemic. Despite their contextual differences (or because of them), our results indicate that some dashboards fulfill their function of communicating, informing decision-making, and supporting behavior change better than others. Moreover, while it is also clear there is no single approach to developing a dashboard, our results suggest that introducing certain features may enhance the actionability of a dashboard.

Knowing the audience and their information needs was identified as a key actionability feature, which corresponds with the Lasswell model for effective communication ([1,41]; Barbazza et al, unpublished data, 2021). However, clear reporting of a dashboard’s purpose (its “why”) and audience (for “whom”) was infrequent. This may be explained in part by the fact that the majority of the dashboards were developed by public authorities and hosted on existing web pages. Hence, the target audience (citizens) and the aim (constitutional mandate to protect health) may be considered implicit. However, without clarity on the intended use and user of a dashboard, its development is steered by the *potential* to be useful rather than addressing a specific information need [32,93-95].

“What” a dashboard communicates through its content is not a neutral window into the available data. It is the result of judgment, discernment, and choice [14]. The average of 5 indicator themes reported per dashboard can be considered to be a manageable volume and is in line with the evidence that “less is more” [33,47]. It is the breadth of types of information presented that is concerningly narrow, with only a handful of dashboards addressing the WHO-recommended four types of information needed for a complete picture of the pandemic [3]. For example, indicators reporting on population behavioral insights gauge the compliance of citizens with infection control measures; thus, they are an important tool for maintaining public trust. However, in our sample, this type of information was rarely reported. This may be due to data infrastructure limitations and the limited availability of these data, especially in the early phases of the pandemic. Similarly, less than half of the dashboards reported on health system management indicators, despite the importance of these indicators in informing the management of both COVID-19 and non–COVID-19 services. Dashboards that did report on these non-epidemiological types of information may serve as inspiration for drawing on innovative data sources and indicators [28,60].

Clarity around data sources and indicator calculations (metadata) are critical for overall quality, credibility, and trustworthiness of reporting [46,48,49]. For transparency on how data were collected and insights into “what lies behind” the reported indicators, providing explicit data sources and calculations should be considered a minimum requirement. Nonetheless, our findings signal that these provisions are not a given. Further efforts are needed internationally and nationally to standardize indicator calculations and set requirements of what constitutes good practice in public reporting of pandemic-related data.

In terms of “how” content is presented, dashboards should be viewed as tools for making clear links between current trends and past policy decisions and individual behavior. Doing so connects change-points and actions, which has been found to contribute to an indicator’s use [96,97]. It also serves to leverage the two-way communication potential of dashboards. Dashboards that fail to make the connection between the past and present miss the opportunity to communicate the effects of users’ decision-making back to them. Beyond describing the past and present, only a handful of dashboards went further and employed predictive analytics by illustrating different future scenarios of “what could happen.” The lack of precision of predictive models and simulations early in the pandemic likely stunted their use. Use of both descriptive and predictive approaches to dashboard design and tighter links between infection control policies and their effects should be further explored into the next phases of the pandemic.

We found frequent use of different display options and interactive techniques among the dashboards assessed. However, the analysis of data by location and by population subgroups was limited overall, which may restrict their utility for individual-level decision-making purposes. The challenge to report data locally and disaggregate the data by relevant breakdowns such as age, sex, socioeconomic status, and ethnic or racial groups may be in large part due to data infrastructure limitations and perceived legal obstacles [98]. Without collecting, registering, and using data about meaningful population subgroups, there is a risk of not being informed about these important (and modifiable) differences [98].

Finally, an actionable dashboard is based on complete, timely, and transparent data that is prepared, contextualized, and presented so that it can be used as information [99]. Our assessment found an overall underuse of known and proven delivery techniques, in particular, the use of explanatory narratives. Plain language text to clarify complicated information has proven to make end users more motivated and confident in using information in their decision-making [1,47,54]. Although commonly used software for the development of dashboards (eg, ArcGIS) has served to optimize their single-screen design, the embedding of narratives into templates may be useful for improving interpretation.

Future research could explore the following points. First, recognizing the highly dynamic nature of COVID-19 dashboards, a follow-up study could provide insights into how dashboards have evolved over time, given improvements in disease prevention, testing, and treatment as well as data infrastructure. Second, exploring across official municipal, regional, and national dashboards in a given context was beyond the scope of this study; however, such an exploration may offer insights into the possibility of tailoring dashboards at different levels to specific purposes and audiences. Third, this study has pursued a theoretically informed expert-based appraisal of actionability. A study from the perspective of the target audience is therefore merited and needed to obtain insights from firsthand use. Finally, the developed assessment tool could be used within a specific country context to analyze actions needed to implement the identified features.

### Strengths and Limitations

To our knowledge, this is the most comprehensive summary of COVID-19 dashboards and assessment of their actionability published to date. The search for COVID-19 dashboards was wide-reaching and used multiple methods to amass a global sample. The approach tapped into a unique and highly specialized international network dedicated to health care performance intelligence, allowing for an expert, context-aware, and multicultural team. The multilanguage competencies of the panel made it possible for the dashboards to be reviewed in their original languages for high-quality data extraction. Through detailed data extraction and a structured process of scoring with joint deliberation, we have identified a set of timely and pragmatic features for optimizing dashboards. This is also the first study to our knowledge on the use of dashboards for public reporting from a communication and health care performance intelligence perspective. Importantly, the study was conducted at pace with the ongoing COVID-19 pandemic to ensure the potential for findings to inform the continued development of dashboards in combination with other communication tools.

We acknowledge the following potential limitations. First, the sample of dashboards is ultimately a subset of publicly available web-based COVID-19 reporting. The sample is also skewed to locations in the European and Pan-American regions, which account for two-thirds of the dashboards reviewed. This can be attributed in part to factors including the thorough but not exhaustive sampling strategy applied; the exclusion of dashboards beyond the 22 language competencies of the panel (ie, Arabic and Hindi); and the focus on web-based dashboards to the exclusion of those exclusively on mobile apps (common to Asian countries). As an exploratory study, reasonable diversity of locations, in combination with different levels (scales) of focus and types of organizations, took precedent and was achieved. Nonetheless, the findings may not be generalizable to all contexts. Second, despite our best efforts to obtain a snapshot of COVID-19 dashboards in a common 2-week period, the severity and specific phase of the pandemic inevitably varied greatly on the date of the review as described. Our approach to assess rather than evaluate the impact of COVID-19 dashboards mitigates the significance of these differences on our findings. Third, the appraised actionability of the dashboards ultimately does not confirm their use in practice, and evaluating this was beyond the scope of this study.

### Conclusion

This study has taken stock of the vast landscape of public web-based COVID-19 dashboards; this is a testament to the advancements in health information systems and digitalization of our societies, coupled with the responsibility and imperative to publicly report health information. As could be expected, the 158 dashboards in our sample, spanning a total of 53 countries, are diverse. They have different contexts and levels of focus, purposes, and audiences. They draw from various data sources, offer different content and use a range of ways—albeit at times limited—to break down data and to visualize, simplify, and interact with information. Their actionability also differs, signaling that their fitness for use by decision makers is not a guarantee. The number of dashboards appraised as highly actionable in the period of July 2020 when the dashboards in this study were assessed signals that work is still needed to optimize the use of dashboards. There is no one-size-fits-all template or model to accomplish this. Dashboards must be purpose-driven and context-specific. We urge those working on COVID-19 dashboards to consider the seven features identified in our study and adopt them as called for. By doing so, they stand to fully leverage the potential advantages of public reporting and its use for decision-making and behavior change needed to address the current pandemic.

## Appendix

Multimedia Appendix 1 Dashboard sampling survey.

Multimedia Appendix 2 Assessment tool.

Multimedia Appendix 3 Public web-based COVID-19 dashboards assessed.

Multimedia Appendix 4 Illustrative indicator titles by theme.

Multimedia Appendix 5 Summary of dashboard scoring.

## Abbreviations

NGT: nominal group technique
WHO: World Health Organization
