# Supplementary material for: Features Constituting Actionable COVID-19 Dashboards: Descriptive Assessment and Expert Appraisal of 158 Public Web-Based COVID-19 Dashboards
Source: J Med Internet Res. 2021 Feb 24;23(2):e25682. doi: 10.2196/25682 (PMC7906125; doi:10.2196/25682)
Supplement: Multimedia Appendix 1 [file jmir_v23i2e25682_app1.docx]

**Multimedia Appendix 1**

Dashboard Sampling Survey

**Title**

Local, regional, national and international web-based reporting on the COVID-19 pandemic: preliminary mapping exercise

**Instructions**

The aim of this exercise is to collect as many websites, using your (local) knowledge and expertise, which we will later assess for content, interpretability and actionability.

You are kindly asked to fill in this form with information on websites which you are aware of that are doing public reporting on the COVID-19 pandemic data at the national level, but also local, regional and international level.

Begin by first filling in those you know about (and follow or even work on) in countries where you currently live and/or originate from.

Continue with other websites you might know about in other countries as well as international ones providing comparative reporting.

Complete a new submission for each dashboard.

**Questions**

1. **Name**

Your name for follow-up as needed.

1. **Link to website**

URL to main dashboard page.

1. **Comments**

Briefly, how would you describe this dashboard? Is it for local, regional, national or international use? In which country? Who is doing the reporting? Any additional comments?
