# Supplementary material for: Features Constituting Actionable COVID-19 Dashboards: Descriptive Assessment and Expert Appraisal of 158 Public Web-Based COVID-19 Dashboards
Source: J Med Internet Res. 2021 Feb 24;23(2):e25682. doi: 10.2196/25682 (PMC7906125; doi:10.2196/25682)
Supplement: Multimedia Appendix 2 [file jmir_v23i2e25682_app2.docx]

**Multimedia Appendix 2**

Assessment Tool

| **Considerations** | **Question** | **Answer options** | **Description** | **Rationale** |
| --- | --- | --- | --- | --- |
| **General** |  |  |  |  |
| Reviewer | Who is reviewing the webpage? | First name | Name of the panel member reviewing the dashboard. | For a record of who the page has been reviewed by. |
| Date of review | When was the page reviewed? | dd-mm-yyyy | Date the review was conducted. | To track the date the page was reviewed. |
| Webpage link | What is the website reviewed? | Add link | This link should direct specifically to the dashboard page. In some instances that is a standalone website, in others it may be found within a specific organization's page. | For referencing purposes. |
| Dashboard archive | Have you archived the dashboard page? | Visit: http://archive.vn/ | During the assessment, "archive" the main page of the dashboard, using http://archive.vn/ and provide the http://archive.vn/xxxx link in this answer field. | To ensure a record of the dashboard on the date of the review is stored for referencing purposes as websites may change regularly. |
| Language of review | What language did you review the webpage in? | Specify language | Reviewers are encouraged to review the dashboard in their most proficient language. This is in addition to the question that follows with regard to the languages in which the dashboard is available. | To record the language in which the assessment and data extract is completed. |
| **Context** |  |  |  |  |
| Level | What is the primary level (scale) of reporting? *Select one* | International, specify  National, specify country Regional (provincial, state, county), specify and country Municipal (city, district), specify and country Other, specify | *International* refers to a multi-country webpage and the range of countries that make up the multiple countries in focus should be specified (e.g. European Union, WHO African Region, South-east Asia, etc.). *National* refers to a specific country – specify the country name. *Regional* refers to the first level sub-national administrative division such as provinces, states, counties ­– specify the specific region of focus. *Municipality* is the second level administrative division such as a city or district – specify the name and country. Other, please specify. | This question aims to assess the primary scale of the webpage and not necessarily the type of analysis. For example, a webpage on Croatia may include international reporting from nearby countries but its primary level is national reporting on Croatia. |
| Organization | Who (which) organization developed the webpage? | Full name | The full name of the organization responsible for the development of the dashboard. If in doubt, check the copyright of the website or contact details. Avoid abbreviations. Extract full name details if an independent, individual-led initiative. | To know who has developed the dashboard. |
| Type of organization | What is the organization type?  *Select one* | International organization (e.g. WHO, OECD, EU)  Governmental (e.g. Government of Canada) Academia (e.g. John Hopkins University) News/media outlet (e.g. Financial Times)  Other (specify) | Select the relevant organization type. *International organizations* have a remit established in agreement with respective member states. *Governmental* is a government or state agency that exercises political authority for a specific jurisdiction be it national or subnational. *Academia* refers to scholarly organizations concerned with research and education. *News/media outlets* refers to publication or broadcast programs that feature stories through media such as newspapers, radio, television, the internet. Other, refers to any organizations that do not meet the above categories and may include individual-led initiatives or newly established groups. | This question assesses the type of organization that has developed the dashboard. The clusters may allow for analysis on differences between types of organizations. The authority of a website’s owner has been found to increase trust and perceived credibility of the information [2]. Differences between organization type will be explored. |
| Language(s) | What are the languages the dashboard is available in and the level of completeness in each? | List, note if equivalent or partially equivalent in each | Consider (if the dashboard is available in more than one language) is the level of information equivalent in each? It may be the case only some text in other languages is translated, rather than the full page. Fully equivalent means all website content is available in an alternative language, including figures. Partially equivalent means only some of the website is available in another language, be it the text or figures. | To determine the range of languages the website is available in and the extent to which it is comparable in each. |
| Scope | What information does the dashboard provide or redirects to*?  *Select all that apply* | Epidemiological info Infection control measures Health system management  Population behavioral insights Social and economic implications  Other (specify) | This question aims to gauge the range of information that is found on the dashboard or can be accessed one click away. While the dashboards predominately will provide epidemiological information, they can also be used as a resource for other types of information*.* These types include: *epidemiological information* relates to the transmission of COVID-10; *health system management* – information relating to the management of health system resources and non-COVID-19 services; *population behavioral insights* such as psychological, societal and cultural factors, and *social and economic implications* such as measures to support families and businesses in response to COVID-19. Select all types of information found on the dashboard reviewed, or *other* if information further to the above are listed. | WHO has identified four key types of information for responding to the pandemic [3]. A focus solely on the epidemiological context can perpetuate a narrow biomedical approach to decision-making and hinder an integrated whole-of-society approach to setting priorities [3,46]. This question aims to gauge to what extent a dashboard reports the core different types of information. |
| **Why** |  |  |  |  |
| Purpose | Is the purpose (*why* the dashboard was developed) mentioned? *Select one* | Yes, please specify No | An explicit purpose of use is defined as the clear statement of the intended decision-making task and in effect, information need, that the dashboard aims to meet [30-32]. Does the website/dashboard explain why it was developed? *Why* (the aim/purpose) a dashboard was developed (e.g. to inform locals about the status of the COVID-19 outbreak in order to take the necessary precautions and be aware of changing risks) is different from a description as to *what* it is about (e.g. a dashboard reporting about COVID-19 in the city of Toronto). If there is a mention of the website's purpose, extract this in full (in English). An example of a clear purpose as to why a dashboard was developed includes: "I created this site and donate my time to update it each day because I want to keep people informed, stop the virus and above all, I believe in the power of data." | A clear purpose of use is central to the actionability of data [43] If the goal of reporting is undefined, the reporting may diminish in effect [1]. |
| Audience (user) | Is the intended audience mentioned (who the dashboard is for) mentioned? *Select one* | Yes, please specify No | If the website explicitly states the target audience, the response is “Yes” and the text where this is stated, should be copied in English. If it is not clear the response is "No". | To be actionable, public reporting should be understood by the intended audience. In order to achieve this, the intended audience of the public reporting should be known in order to address the information needs and priorities of this group [47]. |
| **What** |  |  |  |  |
| Content | Which indicators are reported on the dashboard? | List all indicator titles and their variations (e.g. per day, past 7 days, cumulative) | This question aims to extract the full range of indicator titles available in the dashboard. The varied ways in which the data can be disaggregated (geographically and by different population subgroups) is assessed separately as the range of break downs to follow (e.g. the possibility to disaggregate by sex, age, etc.). | The selection of health care performance indicators and extent to which they respond to the information need is central to fitness for use [43]. The amount of information presented is also of importance, with evidence that “less is more” when presenting the public with information to make choices in health care [33,48]. |
| Data | Is the source of data explicitly noted? *Select one* | Yes, specify No | Select "Yes" if the sources of data are listed explicit either by indicator or for the page in general. These sources may include government databases, national epidemiological institutes, registries, etc. If no data source is explicitly mentioned, select "No". | How and from where data is accessed is a key consideration of use and aspects related to its quality, completeness, and aspects of trustworthiness [49]. |
| Data | Is the metadata specified? *Select one* | Yes No | Select "Yes" if there are supplementary details (e.g. as notes, footnotes, a linked additional webpage) that provide further information on the calculation of the indicators reported. Note, metadata is not 'data caveats' that provide information on how to interpret the quality of the data and what it may or may not include. This captured under 'interpretability'. | An indicator’s level of standardization has known implications for the comparability and analysis of indicators [50]. Differences in the standardization of information (e.g. testing, definitions of mortality and case calculations, varied practices to code COVID-19 related deaths) can lead to inaccurate comparisons when the approach to calculations are not clearly defined. The methodology for gathering data and calculating measures are critical to the accuracy, creditability and understandability of public reporting [47]. |
| Data | How often is the data updated? Is the frequency of updates stated explicitly? | Describe | Take note of a time stamp or explicit mention of data updating frequency (e.g. daily at 6:00 am). | The time-sensitive nature of indicators is a key predictor of use [43]. Time lags in gaining access to data can be a key technical barrier to meaningful action [5]. |
| **How** |  |  |  |  |
| Analysis | Does the analysis include a time trend? If yes, at which interval(s)?  *Select all that apply* | By day  By week  By month No time trend Other (please specify) | This question gauges the time scale used to report the data, e.g. changes per day, per week, per month. Please be sure to note all intervals of time used to present and/or aggregate data, and not simply the most granular scale used for a time trend. | The time interval used in analyzing data has been found a key consideration for managing an indicator’s actionability [43]. The use of a time trend and interval of reporting should be informed by the indicator’s intended purpose of use and users. |
| Analysis | What are the levels (scales) of analysis used? *Select all that apply* | International (multi-country) National (country)  Regional (province, state, county) Municipal (city, district) Neighborhood/post code Other (please specify below) | This question gauges the levels of analysis made. E.g. A national dashboard may report information on the country (national averages), state (subnational averages), and internationally (figures of comparable countries). *International* refers to an analysis across countries. *National* refers to a specific country. *Regional* refers to the first level sub-national administrative division such as provinces, states, counties. *Municipality* is the second level administrative division such as a city or district. *Neighborhood* is the third level of administration division and is typically defined by post codes. If other, specify. | The level (scale) of analysis has been found a key consideration for managing an indicator’s actionability. The level to which an indicator can be disaggregated can serve as a tailoring strategy, making data more relatable to the decision-making. |
| Analysis | What breakdowns are possible? *Select all that apply* | Sex Age Race Ethnicity Long-term care facilities Healthcare workers (incl. long-term care workers) Mode of transmission (incl. imported and/or locally acquired)  Comorbidities Socio-economic status None of the above Other (please specify below) | This question gauges the level of granularity of comparisons used in the analysis. Select the breakdowns possible for the dashboard in general. For example, a breakdown by sex may be possible for all indicators but by age only one. In this example, both age and sex should be selected as possible breakdowns as it is the possibility to assess indicators by different types of breakdowns overall that is being considered. | Disaggregating data can serve as a tailoring strategy, easing decisions by making data more relatable, for example, allowing a user to relate to a particular age cohort or ethnic group [33]. Tailoring of information has been shown to be more effective than generic, high-level information [33,51]. |
| Visualization | How is the data visualized?  *Select all that apply* | Maps Graphs/charts Tables Video/animations None of the above Other (please specify) | Take note of the way in which information is presented. *Maps* include any presentation of a land area to illustrate indicator values. *Graphs/charts* include all variations of graphs/charts, such as bar graphs, trend lines, bubble graphs, demographic graphs, etc. used to present indicator values. *Tables* include displays of day in rows and columns. *Video/animation* includes any display that is automated to be "played" to illustrate trends. Select other for all types of visualizations that do not fit the above categories but did convey information on the identified indicators. | Studies have found there is large variation in the way in which information is publicly reported [16]. A systematic review of consumers’ understanding of online health performance information found that graphs were more effective in communicating quality reports than text of numbers [52]. |
| Interpretation | Is interpretation of the quality and/or meaning of the data guided by contextualizing text? | Yes, to clarify the quality of the data (describe)  Yes, to clarify the meaning of the data (describe)  No | The degree of 'storytelling' can inform the interpretation of findings and support an understanding of what is meant and its caveats [49,54,101]. If a website provides explanation of the *quality of the data,* e.g. potential differences in numbers or figures, or states how or why data is unavailable or unreliable, this should be judged as a “yes” for supporting correct interpretation of data quality. Include an example. If a website provides an explanation of what the *analysis* signals, e.g. this number going down means that there are less cases each day, this should be judged as a "yes" for supporting interpretation of the meaning of the data. Note: Metadata (stating how an indicator was designed or how data was collected) should not be taken into account as interpretative text. (See previous question on metadata) | The use of plain language to clarify the positive direction of an indicator (e.g. a high score is better) has been found to be effective to support the interpretation of complex information [48,55]. End users have been found to be more motivated and more likely to use and comprehend reporting when cognitive effort is reduced and the meaning of data is highlighted [33]. Indicators may be intangible and unfamiliar to users, making it unclear whether a high or a low rate is desirable [1]. |
| Simplicity | Are simplification techniques used to support the interpretation of data? | Use of color coding (e.g. traffic lights) Size variation (e.g. radius of circle to show significance) Icons (e.g. thumbs up, smiles, check mark for good) Other, specify | Simplification techniques are distinct from those that are related to the display (map, table, chart etc.) itself and are rather supplementary to the selected display to support the interpretation of the data by signaling a positive or negative result, trends, etc. Note: the use of colors should be specific to inform the meaning, as in one color signals better performance than another, and not simplify the use of colors for differentiating purposes. | To improve interpretation, simplification techniques can be applied [56]. As the complexity of information is lowered, users have a better understanding and ability to make informed choices [52]. The use of simplification techniques has been found to be more effective than approaches that rely on consumers to make sense of information on their own [52]. |
| Interactiveness | Is the data published in an interactive way? If yes, what does this interaction provide? *Select all that apply and describe* | More information, describe (e.g. clicking on a map/graph, additional info appears)  Change of information, describe (e.g. possible change a graph from cases to deaths) Change of display, describe (e.g. move from graph to map, different type of graph etc., adjusted focus on a graph using a sliding tool)  Other, describe No interactions possible | If the dashboard provides the option to change an indicator's visualization, this is considered 'interactive'. Consider if any of the listed types of interactions are possible anywhere on the dashboard. For each observed, describe what the interaction. | Personal preferences and information needs may differ by viewer, making the degree of flexibility in reporting, where users can choose more details or a summary based on their preferences preferable [52]. Interactive features in online reporting can also increase the trust and perceived credibility of health information reporting websites [2]. |
| **Actionability** |  |  |  |  |
| Actionability | In your expert opinion, how actionable is the dashboard? (for the intended audience or assume general public) | Please elaborate. Consider each of the considerations above with regards to what, why and how the dashboard provides information. | Consider, based on the responses above to describe the dashboard, is the dashboard fit for purpose and use by the intended audience and/or citizens (be it a specific country, region, city, or internationally)? Where citizen refers to a lay member of the public. Provide as much detail as possible to explain your interpretation on whether or not this dashboard is a useful (actionable) resource to inform their decision-making. Be sure to reflect on the dashboards in context. For example, an international dashboard should provide information for a range of countries, whereas a local dashboard should provide information on a very local level. | This appraisal of actionability is an input to a subsequent round of scoring following the further calibration of scoring among the panel of reviewers. The dashboards scored more favorably (5s) will be assessed to distill their common features. In this way, this scoring is a means to an end. |
| Overall score | Overall, on a scale of 1 to 5, how would you rate the actionability of this dashboard?  (1=not actionable; 5=extremely) | 1=Not actionable  2=Slightly actionable  3=Moderately actionable  4=Very actionable  5=Extremely actionable | Based on your reflection above, on a scale of 1 to 5, how would you rate the actionability of the dashboard? Make an initial score for each website as you review each and once you have finalized all your assigned dashboards to review, revisit this score. Is it clear out of the websites reviewed, which was must useful? Does your scoring reflect that? Note: we will review this scoring in a panel exercise to follow. | This appraisal of actionability is an input to a subsequent round of scoring following the further calibration of scoring among the panel of reviewers. The dashboards scored more favorably (5s) will be assessed to distill their common features. In this way, this scoring is a means to an end. |
