# Supplementary material for: Features Constituting Actionable COVID-19 Dashboards: Descriptive Assessment and Expert Appraisal of 158 Public Web-Based COVID-19 Dashboards
Source: J Med Internet Res. 2021 Feb 24;23(2):e25682. doi: 10.2196/25682 (PMC7906125; doi:10.2196/25682)
Supplement: Multimedia Appendix 3 [file jmir_v23i2e25682_app3.docx]

**Public Web-Based COVID-19 Dashboards Assessed**

| **#** | **D-ID** | **Country^*^**  (subregion if applicable) | **Level** | **Archive** | **Organization** | **Review date dd.mm.yyyy** | **COVID-19**  Total on review date | |
| --- | --- | --- | --- | --- | --- | --- | --- | --- |
|  |  |  |  |  |  |  | **Case** | **Deaths** |
| 1 | 77 | [Andorra](https://www.govern.ad/coronavirus) | National | <http://archive.vn/qs3vr> | Government of Andorra | 17.07.2020 | 855 | 52 |
| 2 | 01 | [Armenia](https://ampop.am/covid19-coronavirus-dynamic-statistics-in-armenia/) | National | <http://archive.vn/NPTH7> | Ampop Media | 21.07.2020 | 34,462 | 631 |
| 3 | 57 | [Australia](https://covidlive.com.au/) | National | <http://archive.vn/VtvRw> | COVID Live | 16.07.2020 | 10,810 | 113 |
| 4 | 67 | [Australia](https://www.covid19data.com.au/) | National | <http://archive.vn/CVeo8> | Anthony Macali (Independent) | 08.07.2020 | 8,872 | 106 |
| 5 | 95 | [Australia](https://www.abc.net.au/news/2020-03-17/coronavirus-cases-data-reveals-how-covid-19-spreads-in-australia/12060704?nw=0) | National | <http://archive.vn/o15kK> | ABC News | 17.07.2020 | 11,042 | 116 |
| 6 | 120 | [Australia](https://www.health.gov.au/news/health-alerts/novel-coronavirus-2019-ncov-health-alert) | National | <http://archive.vn/fQSn7> | Australian Government, Department of Health | 06.07.2020 | 8,586 | 106 |
| 7 | 50 | [Australia (Queensland)](https://www.qld.gov.au/health/conditions/health-alerts/coronavirus-covid-19/current-status/statistics) | Regional | <http://archive.vn/WxSRF> | Queensland Government | 17.07.2020 | 1,071 | 6 |
| 8 | 28 | [Australia (Victoria)](https://www.dhhs.vic.gov.au/victorian-coronavirus-covid-19-data) | Regional | <http://archive.vn/9Moo0> | Health and Human Services, Victoria State Government | 07.07.2020 | 2,824 | 22 |
| 9 | 128 | [Austria](https://info.gesundheitsministerium.at/) | National | <http://archive.vn/pAdn0> | Bundesministerium für Soziales, Gesundheit, Pflege und Konsumentenschutz | 16.07.2020 | 19,213 | 706 |
| 10 | 148 | [Belarus](http://stopcovid.belta.by/) | National | <http://archive.vn/ufrRR> | Ministry of Health of Belarus | 17.07.2020 | 65,782 | 491 |
| 11 | 129 | [Belgium](https://epistat.wiv-isp.be/Covid/covid-19.html) | National | <http://archive.vn/px6m3> | Sciensano | 16.07.2020 | 63,039 | 9,675 |
| 12 | 29 | [Bosnia and Herzegovina](https://www.covid-19.ba/) | Regional | <http://archive.vn/8xm6D> | Government of the Federation of Bosnia and Herzegovina, Bosnia and Herzegovina | 07.07.2020 | 2,891 | 68 |
| 13 | 87 | [Brazil](https://covid.saude.gov.br/) | National | <http://archive.today/FtAC6> | Ministry of Health of Brazil | 12.07.2020 | 1,839,850 | 71,469 |
| 14 | 86 | [Brazil](https://experience.arcgis.com/experience/c20767a343bc42178876b8f39bb004bf) | National | <http://archive.today/dH6lS> | Portal GEO | 12.07.2020 | 1,755,779 | 69,184 |
| 15 | 38 | [Canada](https://resources-covid19canada.hub.arcgis.com/) | National | <http://archive.vn/ujVQi> | Esri Canada | 13.07.2020 | 107,589 | 8,783 |
| 16 | 39 | [Canada](https://health-infobase.canada.ca/covid-19/epidemiological-summary-covid-19-cases.html#a5) | National | <http://archive.vn/DW6Ow> | Government of Canada | 13.07.2020 | 107,590 | 8,783 |
| 17 | 40 | [Canada](https://art-bd.shinyapps.io/covid19canada/) | National | <http://archive.vn/m2dLJ> | Jean-Raul R Soucy and Isha Berry (Independent) | 13.07.2020 | 109,920 | 8,827 |
| 18 | 41 | [Canada](https://newsinteractives.cbc.ca/coronavirustracker/) | National | <http://archive.vn/p7Ix2> | Canadian Broadcasting Corporation | 13.07.2020 | 108,278 | 8,825 |
| 19 | 111 | [Canada](https://covid19tracker.ca/) | National | <http://archive.vn/Lqk6L> | Noah Little (Independent) | 18.07.2020 | 99,537 | 8,104 |
| 20 | 112 | [Canada (Alberta)](https://www.alberta.ca/covid-19-alberta-data.aspx) | Regional | <https://archive.vn/hQ9Kl> | Government of Alberta | 18.07.2020 | 9,114 | 165 |
| 21 | 110 | [Canada (British Columbia)](https://experience.arcgis.com/experience/a6f23959a8b14bfa989e3cda29297ded) | Regional | <http://archive.vn/Tspb3> | Provincial Health Services Authority, BC Center for Disease Control | 18.07.2020 | 3,198 | 189 |
| 22 | 113 | [Canada (Manitoba)](https://experience.arcgis.com/experience/f55693e56018406ebbd08b3492e99771) | Regional | <https://archive.vn/lT4SS> | Government of Manitoba | 18.07.2020 | 273 | 6 |
| 23 | 114 | [Canada (Montreal)](https://santemontreal.qc.ca/en/public/coronavirus-covid-19/situation-of-the-coronavirus-covid-19-in-montreal/#c43674) | Municipal | <https://archive.vn/Dfwbz> | Government of Montreal | 18.07.2020 | 27,863 | 3,431 |
| 24 | 115 | [Canada (New Brunswick)](https://www2.gnb.ca/content/gnb/en/corporate/promo/covid-19.html) | Regional | <http://archive.vn/xEOZl> | Government of New Brunswick | 20.07.2020 | 164 | 2 |
| 25 | 116 | [Canada (Newfoundland and Labrador)](https://covid-19-newfoundland-and-labrador-gnl.hub.arcgis.com/) | Regional | <http://archive.vn/xv4sw> | Government of Newfoundland and Labrador | 20.07.2020 | 262 | 3 |
| 26 | 30 | [Canada (Northwest Territories)](https://www.gov.nt.ca/covid-19/) | Regional | <http://archive.vn/UNPBb> | Government of Northwest Territories | 07.07.2020 | 5 | NA |
| 27 | 58 | [Canada (Nova Scotia)](https://novascotia.ca/coronavirus/data/) | Regional | <http://archive.vn/LOfCa> | Government of Nova Scotia | 16.07.2020 | 1,067 | 63 |
| 28 | 31 | [Canada (Nunavut)](https://www.gov.nu.ca/health/information/covid-19-novel-coronavirus) | Regional | <http://archive.vn/HLobA> | Nunavut Department of Health | 07.07.2020 | 0 | 0 |
| 29 | 42 | [Canada (Ontario)](https://howsmyflattening.ca/#/home) | Regional | <http://archive.vn/7T6MT> | #HowsMyFlattening | 14.07.2020 | 36,950 | 2,722 |
| 30 | 59 | [Canada (Ontario)](https://www.publichealthontario.ca/en/data-and-analysis/infectious-disease/covid-19-data-surveillance/covid-19-data-tool) | Regional | <http://archive.vn/YtJVv> | Ontario Agency for Health Protection and Promotion | 16.07.2020 | 37,052 | NA |
| 31 | 70 | [Canada (Ontario)](https://covid-19.ontario.ca/data) | Regional | <http://archive.vn/1Urge> | Government of Ontario | 09.07.2020 | 36,178 | 2,700 |
| 32 | 130 | [Canada (Ottawa)](https://www.ottawapublichealth.ca/en/reports-research-and-statistics/daily-covid19-dashboard.aspx) | Municipal | <http://archive.vn/3ZL3k> | Ottawa Public Health | 16.07.2020 | 2,167 | 263 |
| 33 | 43 | [Canada (Prince Edward Island)](https://www.princeedwardisland.ca/en/information/health-and-wellness/pei-covid-19-case-data) | Regional | <http://archive.vn/oXtDj> | Government of Prince Edward Island | 13.07.2020 | 33 | NA |
| 34 | 19 | [Canada (Quebec)](https://www.quebec.ca/sante/problemes-de-sante/a-z/coronavirus-2019/situation-coronavirus-quebec/) | Regional | <http://archive.vn/jky19> | Government of Quebec | 14.07.2020 | 55,937 | 5,577 |
| 35 | 20 | [Canada (Quebec)](https://www.inspq.qc.ca/covid-19/donnees) | Regional | [http://archive.vn/D3JfB on 14.07.20http://archive.vn/D3JfB](http://archive.vn/D3JfB%20on%2014.07.20) | National Institute of Public Health of Quebec | 14.07.2020 | 56,730 | 5,633 |
| 36 | 117 | [Canada (Saskatchewan)](https://dashboard.saskatchewan.ca/health-wellness) | Regional | <http://archive.vn/kVUii> | Government of Saskatchewan | 20.07.2020 | 943 | 15 |
| 37 | 44 | [Canada (Toronto)](https://www.toronto.ca/home/covid-19/covid-19-latest-city-of-toronto-news/covid-19-status-of-cases-in-toronto/) | Municipal | <http://archive.vn/NoyJh> | City of Toronto | 14.07.2020 | 14,735 | 1,110 |
| 38 | 118 | [Canada (Vancouver)](https://covid19dashboard.vancouver.ca/) | Municipal | <http://archive.vn/jPIlp> | City of Vancouver | 20.07.2020 | NA | NA |
| 39 | 45 | [Canada (Yukon)](https://yukon.ca/en/case-counts-covid-19) | Regional | <http://archive.vn/cG5a0> | Government of Yukon | 13.07.2020 | 15 | NA |
| 40 | 79 | [Chile](https://www.gob.cl/coronavirus/cifrasoficiales/) | National | <http://archive.vn/FND66> | Chilean Government | 17.07.2020 | 323,698 | 7,290 |
| 41 | 105 | [China](https://news.qq.com/zt2020/page/feiyan.htm#/?nojump=1) | National | <http://archive.vn/2oPeq> | Tencent Holdings Ltd. | 14.07.2020 | 85,671 | 5,649 |
| 42 | 106 | [China](https://wp.m.163.com/163/page/news/virus_report/index.html?_nw_=1&_anw_=1) | National | <http://archive.vn/F8Qqf> | NetEase | 15.07.2020 | 85,696 | 4,649 |
| 43 | 107 | [China](https://ncov.dxy.cn/ncovh5/view/pneumonia) | National | <http://archive.vn/utd4M> | DXY | 17.07.2020 | NA | NA |
| 44 | 138 | [China](http://2019ncov.chinacdc.cn/2019-nCoV/) | National | <http://archive.vn/HGVWY> | Chinese Center for Disease Control and Prevention | 11.07.2020 | 85,487 | 4,648 |
| 45 | 139 | [China](https://news.sina.cn/zt_d/yiqing0121) | National | <http://archive.vn/q7aLz> | Sina News | 17.07.2020 | 85,840 | 4,652 |
| 46 | 140 | [China](https://voice.baidu.com/act/newpneumonia/newpneumonia) | National | <http://archive.vn/Tg14i> | Baidu Inc. | 17.07.2020 | 85,840 | 4,651 |
| 47 | 09 | [Costa Rica](http://geovision.uned.ac.cr/oges/) | National | <http://archive.today/2020.07.14-100448/http://geovision.uned.ac.cr/oges/> | Costa Rican Ministry of Health, Geographical Health Observatory | 13.07.2020 | 8,036 | NA |
| 48 | 10 | [Costa Rica](https://oddapp2.shinyapps.io/CoronavirusCostaRica/) | National | <http://archive.today/2020.07.14-100758/https://oddapp2.shinyapps.io/CoronavirusCostaRica/> | University of Costa Rica | 13.07.2020 | 8,036 | NA |
| 49 | 80 | [Costa Rica](https://www.arcgis.com/apps/webappviewer/index.html?id=5bebb786f1a74912accec9bb2d341ce8&extent=-9633666.8308%2C870866.4684%2C-9115118.0309%2C1380854.3211%2C102100) | National | <https://archive.vn/8Spsq> | Escuela de Ciencias Geográficas  Universidad Nacional | 17.07.2020 | 9,546 | NA |
| 50 | 21 | [Cote d’Ivoire](https://covid19-ci.info/) | National | <http://archive.vn/nnYbl> | AFRIX | 17.07.2020 | 13,554 | 87 |
| 51 | 31 | [Croatia](https://www.koronavirus.hr/) | National | <http://archive.vn/pRybd> | Government of the Republic of Croatia | 07.07.2020 | 3,272 | 113 |
| 52 | 46 | [Denmark](https://www.ssi.dk/sygdomme-beredskab-og-forskning/sygdomsovervaagning/c/covid19-overvaagning) | National | <http://archive.vn/zzVm9> | State Serum Institute | 13.07.2020 | 13,037 | 610 |
| 53 | 96 | [Denmark](https://www.sst.dk/da/corona/tal-og-overvaagning) | National | <http://archive.vn/9rlyG> | Danish Health Authority | 17.07.2020 | 13,124 | 610 |
| 54 | 81 | [Estonia](https://koroonakaart.ee/en) | National | <http://archive.vn/XxB8q> | Independent | 17.07.2020 | 2,016 | 69 |
| 55 | 141 | [Finland](https://experience.arcgis.com/experience/92e9bb33fac744c9a084381fc35aa3c7) | National | <http://archive.vn/lznaE> | Finnish Institute for Health and Welfare (THL) | 17.07.2020 | 7,361 | NA |
| 56 | 22 | [France](https://www.gouvernement.fr/info-coronavirus/carte-et-donnees) | National | <http://archive.vn/7O2ib> | Government of France | 15.07.2020 | NA | NA |
| 57 | 23 | [France](https://geodes.santepubliquefrance.fr/#view=map2&c=indicator) | National | <http://archive.vn/nhsHy> | Santé Publique France (Public Health France) | 16.07.2020 | NA | NA |
| 58 | 24 | [France](https://coronavirus.politologue.com/) | National | <http://archive.vn/E8RSW> | Politologue.com | 16.07.2020 | 213,302 | 30,138 |
| 59 | 132 | [Germany](https://experience.arcgis.com/experience/478220a4c454480e823b17327b2bf1d4) | National | <http://archive.vn/wQLbJ> | Robert Koch-Institut | 16.07.2020 | 200,260 | 9,078 |
| 60 | 135 | [Germany](https://interaktiv.morgenpost.de/corona-virus-karte-infektionen-deutschland-weltweit/) | National | <http://archive.vn/JF9dW> | Berliner Morgenpost | 16.07.2020 | 201,450 | 9,087 |
| 61 | 131 | [Germany (Bavaria)](https://www.lgl.bayern.de/gesundheit/infektionsschutz/infektionskrankheiten_a_z/coronavirus/karte_coronavirus/index.htm) | Regional | <http://archive.vn/W0ASm> | Bayerisches Landesamt für Gesundheit und Lebensmittelsicherheit | 16.07.2020 | 49,522 | 2,610 |
| 62 | 133 | [Germany (Hamburg)](https://www.hamburg.de/coronavirus/) | Municipal | <http://archive.vn/9gDMO> | City of Hamburg | 16.07.2020 | 5,231 | NA |
| 63 | 47 | [Greenland (Kingdom of Denmark)](https://nun.gl/Emner/Borgere/Coronavirus_emne/Foelg_smittespredningen?sc_lang=da) | Regional | <http://archive.vn/DV4aX> | Government of Greenland | 13.07.2020 | 13 | 0 |
| 64 | 108 | [Hong Kong](https://chp-dashboard.geodata.gov.hk/covid-19/en.html) | Regional | <http://archive.vn/M70Ve> | Development Bureau, Lands Department, Smart City Consortium | 17.07.2020 | 1,656 | 10 |
| 65 | 88 | [Hungary](https://koronavirus.gov.hu/#/) | National | <http://archive.today/tJcw7> | Government of Hungary | 13.07.2020 | NA | 235 |
| 66 | 51 | [Indonesia](https://covid19.go.id/peta-sebaran) | National | <http://archive.vn/tuJPH> | Government of Indonesia | 17.07.2020 | 83,130 | 3,957 |
| 67 | 53 | [Indonesia](https://kawalcovid19.id/) | National | <http://archive.vn/kV4Je> | KawalCOVID19 | 22.07.2020 | 88,214 | 4,239 |
| 68 | 52 | [Indonesia (Central Java)](https://corona.jatengprov.go.id/data) | Regional | <http://archive.vn/BIMIA> | Government of Central Java | 18.07.2020 | 6,963 | 599 |
| 69 | 92 | [Ireland](https://covid19ireland-geohive.hub.arcgis.com/) | National | <http://archive.today/7araW> | Government of Ireland | 12.07.2020 | 25,638 | 1,746 |
| 70 | 71 | [Italy](http://opendatadpc.maps.arcgis.com/apps/opsdashboard/index.html#/b0c68bce2cce478eaac82fe38d4138b1) | National | <http://archive.vn/1nAq6> | Department of Civil Protection | 09.07.2020 | 35,708 | 35,587 |
| 71 | 76 | [Italy](https://www.epicentro.iss.it/coronavirus/sars-cov-2-dashboard) | National | <http://archive.vn/JtfvM> | Italian National Institute of Health | 10.07.2020 | NA | NA |
| 72 | 150 | [Kazakhstan](https://www.coronavirus2020.kz/) | National | <http://archive.vn/ayFmB> | Kazinform | 17.07.2020 | 66,895 | 374 |
| 73 | 151 | [Kazakhstan](https://hls.kz/) | National | <http://archive.vn/nTuiH> | National Center for Public Health | 17.07.2020 | 63,514 | 375 |
| 74 | 153 | [Kyrgyzstan](http://www.med.kg/ru/informatsii.html) | National | <http://archive.vn/UsuGE> | Ministry of Health of Kyrgyz Republic | 17.07.2020 | 13,101 | 172 |
| 75 | 158 | [Kyrgyzstan](https://mangement4health.maps.arcgis.com/apps/opsdashboard/index.html#/ba9465073d374eaca04c891c079a5c46) | National | <http://archive.vn/QPLQo> | Nm4h | 17.07.2020 | 13,101 | 172 |
| 76 | 122 | [Luxembourg](https://coronavirus.gouvernement.lu/en.html) | National | <http://archive.vn/bFcLC> | The Luxembourg Government | 08.07.2020 | 4,603 | 110 |
| 77 | 55 | [Malaysia](http://covid-19.moh.gov.my/) | National | <http://archive.vn/xB8eM> | Government of Malaysia | 22.07.2020 | 6,941 | 113 |
| 78 | 123 | [Malta](https://deputyprimeminister.gov.mt/en/health-promotion/covid-19/Pages/covid-19-infographics.aspx) | National | <http://archive.vn/UBjWp> | Ministry of Health of Malta | 08.07.2020 | 673 | 9 |
| 79 | 14 | [Mexico](https://coronavirus.gob.mx/datos/) | National | <http://archive.today/2020.07.16-230211/https://coronavirus.gob.mx/fHDMap/mun.php> | Government of Mexico | 16.07.2020 | NA | NA |
| 80 | 16 | [Mexico](https://covid19.sinave.gob.mx/) | National | <http://archive.today/2020.07.17-120351/https://covid19.sinave.gob.mx/mapaestimados.aspx> | Ministry of Health of Mexico | 17.07.2020 | 48,857 | NA |
| 81 | 15 | [Mexico (Mexico City)](https://hospitales.covid19.cdmx.gob.mx/public/hospitales/EstatusHospitales.xhtml?tamizajeRealizado=true) | Municipal | <http://archive.today/2020.07.17-103808/https://hospitales.covid19.cdmx.gob.mx/public/hospitales/EstatusHospitales.xhtml?tamizajeRealizado=true> | Ministry of Health of Mexico City | 17.07.2020 | NA | NA |
| 82 | 33 | [Montenegro](https://www.ijzcg.me/) | National | <http://archive.vn/1FC5J> | Public Health Institute of Montenegro | 07.07.2020 | 841 | 14 |
| 83 | 25 | [Morocco](http://www.covidmaroc.ma/Pages/Accueil.aspx) | National | <http://archive.vn/SC0pu> | Ministry of Health | 17.07.2020 | 16,638 | 263 |
| 84 | 124 | [Netherlands](https://www.rivm.nl/coronavirus-covid-19/actueel) | National | <http://archive.vn/7O4Z7> | National Institute for Public Health and the Environment (RIVM) | 08.07.2020 | NA | NA |
| 85 | 125 | [Netherlands](https://www.nivel.nl/nl/nivel-zorgregistraties-eerste-lijn/monitor-cijfers-covid-19-huisartsenpraktijken) | National | <http://archive.vn/GSsQG> | The Netherlands Institute for Health Services Research (Nivel) | 10.07.2020 | NA | NA |
| 86 | 137 | [Netherlands](https://coronadashboard.rijksoverheid.nl/) | National | <http://archive.vn/lBh0Q> | Rijksoverheid | 16.07.2020 | NA | NA |
| 87 | 61 | [New Zealand](https://nzcoviddashboard.esr.cri.nz/#!/) | National | <http://archive.vn/MNW86> | New Zealand Environmental Science and Research Institute | 17.07.2020 | 1,549 | 22 |
| 88 | 62 | [New Zealand](https://www.health.govt.nz/our-work/diseases-and-conditions/covid-19-novel-coronavirus/covid-19-current-situation/covid-19-current-cases) | National | <http://archive.vn/zpKDU> | New Zealand Ministry of Health – Manatū Hauora | 17.07.2020 | 1,548 | 22 |
| 89 | 63 | [New Zealand](https://www.stats.govt.nz/experimental/covid-19-data-portal) | National | <http://archive.vn/8Llz2> | Stats NZ | 17.07.2020 | NA | 22 |
| 90 | 100 | [Norway](https://www.helsedirektoratet.no/statistikk/antall-innlagte-pasienter-pa-sykehus-med-pavist-covid-19#datakilde-og-frekvens-paa-oppdatering) | National | <http://archive.vn/yv3FO> | Norwegian Directorate of Health | 17.07.2020 | NA | NA |
| 91 | 101 | [Norway](https://www.fhi.no/sv/smittsomme-sykdommer/corona/dags--og-ukerapporter/dags--og-ukerapporter-om-koronavirus/) | National | <http://archive.vn/ybdAv> | Norwegian Institute of Public Health | 17.07.2020 | 9,011 | 254 |
| 92 | 56 | [Philippines](https://ncovtracker.doh.gov.ph/) | National | <http://archive.vn/fuyCK> | Government of Philippines | 23.07.2020 | NA | NA |
| 93 | 93 | [Portugal](https://covid19.min-saude.pt/ponto-de-situacao-atual-em-portugal/) | National | <http://archive.today/ZqkTI> | Directorate-General of Health | 14.07.2020 | 46,818 | 1,662 |
| 94 | 04 | [Republic of Moldova](http://gismoldova.maps.arcgis.com/apps/opsdashboard/index.html#/d274da857ed345efa66e1fbc959b021b) | National | <http://archive.vn/74MwO> | Ministry of Health, Labour and Social Protection | 20.07.2020 | 20,980 | 688 |
| 95 | 05 | [Romania](https://instnsp.maps.arcgis.com/apps/opsdashboard/index.html#/5eced796595b4ee585bcdba03e30c127) | National | <http://archive.vn/xZCf2> | National Institute for Public Health | 21.07.2020 | 38,139 | 2,038 |
| 96 | 06 | [Romania](https://datelazi.ro/) | National | <https://archive.vn/F6Ap3> | Code for Romania | 20.07.2020 | 38,139 | 2,038 |
| 97 | 07 | [Romania](https://www.mai.gov.ro/informare-covid-19-grupul-de-comunicare-strategica-20-iulie-ora-13-00/) | National | <http://archive.vn/ro9Eq> | Romanian Ministry of Interior | 21.07.2020 | 38,139 | 2,038 |
| 98 | 154 | [Russian Federation](https://xn--80aesfpebagmfblc0a.xn--p1ai/) | National | <http://archive.vn/7sO5i> | Ministry of Health of Russian Federation and Federal Service for Supervision of Consumer Rights Protection and Human Well-Being | 17.07.2020 | 759,203 | 12,123 |
| 99 | 155 | [Russian Federation](https://covid19.rosminzdrav.ru/) | National | <http://archive.vn/lgCmt> | Ministry of Health of Russian Federation | 17.07.2020 | 752,797 | 11,937 |
| 100 | 26 | [Senegal](http://www.sante.gouv.sn/Pr%C3%A9sentation/coronavirus-riposte-%C3%A0-l%C3%A9pid%C3%A9mie-tableau-r%C3%A9capitulatif-des-dons-en-esp%C3%A8ces) | National | <http://archive.vn/SC0pu> | Ministry of Health | 17.07.2020 | 8,544 | 160 |
| 101 | 34 | [Serbia](https://covid19.rs/) | National | <http://archive.vn/4bOrf> | Serbian Ministry of Health and the Institute of Public Health of Serbia “Dr Milan Jovanovic Batut” | 07.07.2020 | 16,719 | 330 |
| 102 | 35 | [Slovakia](https://covid-19.nczisk.sk/en) | National | <http://archive.vn/d5VgU> | Ministry of Health of the Slovak Republic | 07.07.2020 | 1,767 | 28 |
| 103 | 72 | [Slovenia](https://covid-19.sledilnik.org/sl/stats) | National | <http://archive.vn/szM4d> | Sledilnik.org | 09.07.2020 | 1,776 | 111 |
| 104 | 73 | [Slovenia](https://www.nijz.si/sl/dnevno-spremljanje-okuzb-s-sars-cov-2-covid-19) | National | <http://archive.vn/qQlLH> | National Institute of Public Health | 09.07.2020 | 1,776 | 111 |
| 105 | 74 | [Slovenia](https://www.gov.si/teme/koronavirus-sars-cov-2/) | National | <http://archive.vn/eaxEk> | Government Communication Office | 09.07.2020 | NA | NA |
| 106 | 75 | [Slovenia](https://monitor.ustavimokorono.si/) | National | <https://archive.vn/RZlgE> | Matej Kovačič, Tomaž Korenika and Tine Mezgec (Independent) | 09.07.2020 | NA | NA |
| 107 | 144 | [Singapore](https://www.moh.gov.sg/covid-19) | National | <http://archive.vn/64Plt> | Ministry of Health of Singapore | 17.07.2020 | 43,577 | 27 |
| 108 | 145 | [South Korea](https://coronaboard.kr/en/) | National | <http://archive.vn/aMu3a> | Corona Board | 17.07.2020 | 13,672 | 293 |
| 109 | 17 | [Spain](https://cnecovid.isciii.es/covid19/) | National | <http://archive.today/2020.07.17-123630/https://cnecovid.isciii.es/covid19/> | National Center of Epidemiology | 17.07.2020 | NA | NA |
| 110 | 83 | [Spain](https://www.rtve.es/noticias/20200717/mapa-del-coronavirus-espana/2004681.shtml) | National | <http://archive.vn/87f2J> | Corporación de Radio y Televisión Española | 17.07.2020 | 258,855 | 28,416 |
| 111 | 146 | [Sweden](https://portal.icuregswe.org/siri/report/corona.covid-dagligen) | National | <http://archive.vn/fG42h> | Swedish Intensive Care Registry (SIR) | 17.07.2020 | NA | NA |
| 112 | 147 | [Sweden](https://experience.arcgis.com/experience/09f821667ce64bf7be6f9f87457ed9aa) | National | <https://archive.vn/tfGbi> | Public Health Agency of Sweden (Folkhälsomyndigheten) | 17.07.2020 | 77,281 | 5,619 |
| 113 | 36 | [Switzerland](https://www.corona-data.ch/en) | National | <http://archive.vn/LmvZG> | Independent | 08.07.2020 | 1,518 | 11 |
| 114 | 136 | [Switzerland](https://www.bag.admin.ch/bag/de/home/krankheiten/ausbrueche-epidemien-pandemien/aktuelle-ausbrueche-epidemien/novel-cov/situation-schweiz-und-international.html) | National | <http://archive.vn/PbPNU> | Bundesamt für Gesundheit BAG | 16.07.2020 | 33,290 | 1,688 |
| 115 | 27 | [Switzerland (Vaud)](https://www.vd.ch/toutes-les-actualites/hotline-et-informations-sur-le-coronavirus/point-de-situation-statistique-dans-le-canton-de-vaud/) | Regional | <http://archive.vn/9qbXP> | County of Vaud | 16.07.2020 | NA | NA |
| 116 | 119 | [Taiwan](https://sites.google.com/cdc.gov.tw/2019-ncov/taiwan) | Regional | <http://archive.vn/80iSm> | Taiwan CDC | 20.07.2020 | 513 | 5 |
| 117 | 156 | [Tajikistan](https://covid.tj/#cv-charts) | National | <http://archive.vn/DYZCL> | Ministry of Health and Social Protection of the Republic of Tajikistan | 17.07.2020 | 6,786 | 56 |
| 118 | 102 | [Turkey](https://covid19.saglik.gov.tr/) | National | <https://archive.vn/ZJCKw> | Ministry of Health of Turkey | 17.07.2020 | 216,873 | 5,440 |
| 119 | 48 | [United Kingdom](https://clininf.eu/index.php/cov-19/) | National | <http://archive.vn/3Qymk> | University of Oxford and Royal College of General Practitioners | 14.07.2020 | NA | NA |
| 120 | 64 | [United Kingdom](https://coronavirus.data.gov.uk/) | National | <http://archive.vn/q5ilb> | Public Health England | 17.07.2020 | 292,552 | 45,119 |
| 121 | 65 | [United Kingdom](https://www.bbc.com/news/uk-51768274) | National | <http://archive.vn/JLwQH> | British Broadcasting Association | 17.07.2020 | 292,552 | 45,119 |
| 122 | 08 | [United States](https://www.cdc.gov/coronavirus/2019-ncov/cases-updates/index.html) | National | <http://archive.vn/TExg1> | Centres for Disease Control and Prevention | 22.07.2020 | 3,761,362 | 140,157 |
| 123 | 18 | [United States](https://covidtracking.com/data) | National | <http://archive.today/2020.07.16-003040/https://covidtracking.com/data> | The COVID Tracking Project (The Atlantic) | 17.07.2020 | 3,478,419 | 129,595 |
| 124 | 49 | [United States](https://covidactnow.org/?s=58173) | National | <http://archive.vn/7hgsL> | COVID Act Now | 14.07.2020 | NA | NA |
| 125 | 37 | [United States (California)](https://covid19.ca.gov/) | Regional | <http://archive.vn/u41FC> | California State Government | 08.07.2020 | 277,774 | 6,448 |
| 126 | 94 | [United States (Colorado)](https://covid19.colorado.gov/data) | Regional | <http://archive.today/I3yam> | Colorado Department of Public Health and Environment | 14.07.2020 | 37,242 | 1,589 |
| 127 | 126 | [United States (Florida)](https://fdoh.maps.arcgis.com/apps/opsdashboard/index.html#/8d0de33f260d444c852a615dc7837c86) | Regional | <http://archive.vn/ZzNtp> | Department of Health Florida | 10.07.2020 | 232,718 | 4,009 |
| 128 | 103 | [United States (Maryland)](https://coronavirus.maryland.gov/) | Regional | <http://archive.vn/up0WC> | Maryland Department of Health | 17.07.2020 | 75,664 | 3,215 |
| 129 | 104 | [United States (New York State)](https://health.data.ny.gov/Health/New-York-State-Statewide-COVID-19-Testing/xdss-u53e/data) | Regional | <http://archive.vn/hq6td> | New York State | 17.07.2020 | NA | NA |
| 130 | 85 | [United States (New York City)](https://www1.nyc.gov/site/doh/covid/covid-19-data.page) | Municipal | <http://archive.vn/o1Ngx> | NYC Health Department | 17.07.2020 | 217,230 | 18,754 |
| 131 | 66 | [United States (Los Angeles)](http://dashboard.publichealth.lacounty.gov/covid19_surveillance_dashboard/) | Regional | <http://archive.vn/QBXTE> | Los Angeles County Department of Public Health | 17.07.2020 | 139,841 | 3,731 |
| 132 | 127 | [United States (Washington State)](https://www.doh.wa.gov/Emergencies/NovelCoronavirusOutbreak2020COVID19/DataDashboard) | Regional | <http://archive.vn/94G5T> | Washington State Department of Health | 13.07.2020 | 38,581 | 1,409 |
| 133 | 157 | [Uzbekistan](https://coronavirus.uz/ru) | National | <http://archive.vn/LwOPM> | Ministry of Healthcare of the Republic of Uzbekistan | 17.07.2020 | 15,482 | 77 |
| 134 | 121 | [Africa](https://www.afro.who.int/health-topics/coronavirus-covid-19) | International | <http://archive.vn/zIivC> | WHO Regional Office for Africa | 08.07.2020 | NA | NA |
| 135 | 12 | [Europe and Central Asia](https://who.maps.arcgis.com/apps/opsdashboard/index.html#/ead3c6475654481ca51c248d52ab9c61) | International | <http://archive.vn/2020.07.14-130813/https://who.maps.arcgis.com/apps/opsdashboard/index.html%23/ead3c6475654481ca51c248d52ab9c61> | WHO Regional Office for Europe | 14.07.2020 | 2,956,087 | 204,061 |
| 136 | 54 | [South-East Asia](https://www.who.int/southeastasia/outbreaks-and-emergencies/novel-coronavirus-2019) | International | <http://archive.vn/0DLK8> | WHO Regional Office for South-East Asia | 22.07.2020 | 88,214 | 4,239 |
| 137 | 109 | [Western Pacific](https://who.maps.arcgis.com/apps/opsdashboard/index.html#/345dfdc82b5c4f6a815f1d54a05d18ec) | International | <http://archive.vn/N2b8i> | WHO Regional Office for Western Pacific | 17.07.2020 | 254,236 | 7,879 |
| 138 | 02 | [Global](https://ncov2019.live/) | International | <http://archive.vn/UWv29> | Avi Schiffmann (Independent) | 21.07.2020 | 14,867,503 | 613,550 |
| 139 | 03 | [Global](https://visalist.io/emergency/coronavirus) | International | <http://archive.vn/HANQA> | Visa List | 20.07.2020 | 14,510,000 | 606,200 |
| 140 | 11 | [Global](https://coronavirus.1point3acres.com/en) | International | <http://archive.today/2020.07.14-101318/https://coronavirus.1point3acres.com/en> | 1Point3Acres | 14.07.2020 | 13,103,290 | 573,042 |
| 141 | 13 | [Global](http://www.ihi.org/Topics/COVID-19/Pages/COVID-19-Data-Dashboard.aspx) | International | <http://archive.today/2020.07.16-222335/http://www.ihi.org/Topics/COVID-19/Pages/COVID-19-Data-Dashboard.aspx> | Institute for Healthcare Improvement | 16.07.2020 | NA | NA |
| 142 | 60 | [Global](https://www.nytimes.com/interactive/2020/world/coronavirus-maps.html) | International | <http://archive.vn/3govA> | The New York Times | 16.07.2020 | 13,330,200 | 578,800 |
| 143 | 68 | [Global](https://coronavirus.jhu.edu/map.html) | International | <http://archive.vn/SCIfy> | Johns Hopkins University | 08.07.2020 | 11,856,991 | 544,871 |
| 144 | 69 | [Global](https://www.coronatracker.com/analytics) | International | <http://archive.vn/STFQO> | Corona Tracker | 08.07.2020 | 11,995,641 | 547,592 |
| 145 | 78 | [Global](https://www2.deloitte.com/ca/en/pages/about-deloitte/articles/covid-dashboard.html) | International | <http://archive.vn/qrJbw> | Deloitte. | 17.07.2020 | NA | NA |
| 146 | 82 | [Global](https://vac-lshtm.shinyapps.io/ncov_tracker/) | International | <http://archive.vn/nz2oO> | London School of Hygiene and Tropical Medicine | 17.07.2020 | 13,805,296 | 589,911 |
| 147 | 84 | [Global](https://ig.ft.com/coronavirus-chart/?areas=eur&areas=usa&areas=bra&areas=gbr&areasRegional=usny&areasRegional=usca&areasRegional=usfl&areasRegional=ustx&cumulative=0&logScale=1&perMillion=0&values=deaths) | International | <http://archive.vn/RBrv0> | Financial Times | 17.07.2020 | NA | NA |
| 148 | 89 | [Global](https://hgis.uw.edu/virus/) | International | <http://archive.today/uccB1> | Humanistic GIS Lab at University of Washington | 13.07.2020 | NA | NA |
| 149 | 90 | [Global](https://www.accuweather.com/en/pt/national/covid-19) | International | <http://archive.today/UFPKo> | AccuWeather | 13.07.2020 | 12,910,231 | 569,123 |
| 150 | 91 | [Global](https://covid-statistics.jrc.ec.europa.eu/Home/Dashboard) | International | <http://archive.today/g1EGt> | European Commission | 13.07.2020 | NA | NA |
| 151 | 97 | [Global](https://www.worldometers.info/coronavirus/) | International | <http://archive.vn/FC1yH> | Worldometer | 17.07.2020 | 13,967,833 | 593,100 |
| 152 | 98 | [Global](http://www.healthdata.org/covid) | International | <http://archive.vn/KK2cS> | The Institute for Health Metrics and Evaluation | 17.07.2020 | NA | 629,995 |
| 153 | 99 | [Global](http://91-divoc.com/pages/covid-visualization/) | International | <http://archive.vn/fJ9tk> | Prof. Wade Fagen-Ulmschneider (Independent) | 17.07.2020 | NA | NA |
| 154 | 134 | [Global](https://ourworldindata.org/coronavirus) | International | <http://archive.vn/rEwvE> | University of Oxford | 16.07.2020 | NA | NA |
| 155 | 142 | [Global](https://qap.ecdc.europa.eu/public/extensions/COVID-19/COVID-19.html) | International | <http://archive.vn/yPffj> | European Centre for Disease Prevention and Control | 17.07.2020 | 13,788,300 | 589,688 |
| 156 | 143 | [Global](https://covid19.who.int/) | International | <http://archive.vn/Kq0az> | World Health Organization | 17.07.2020 | 13,615,593 | 585,727 |
| 157 | 149 | [Global](https://yandex.by/web-maps/covid19?ll=41.775580%2C54.894027&z=3) | International | <http://archive.vn/lLkqP> | Yandex | 17.07.2020 | 13,044,248 | 577,885 |
| 158 | 152 | [Global](https://rus.ozodi.org/a/30507071.html) | International | <http://archive.vn/Z5WeI> | Radio Ozodi | 17.07.2020 | 13,872,566 | 591,342 |

^*^Country status according to WHO classification <https://www.who.int/choice/demography/by_country/en/>

NA: not available (not specified or available through archiving); D-1D: dashboard identifier
