# Supplementary material for: Features Constituting Actionable COVID-19 Dashboards: Descriptive Assessment and Expert Appraisal of 158 Public Web-Based COVID-19 Dashboards
Source: J Med Internet Res. 2021 Feb 24;23(2):e25682. doi: 10.2196/25682 (PMC7906125; doi:10.2196/25682)
Supplement: Multimedia Appendix 4 [file jmir_v23i2e25682_app4.docx]

**Multimedia Appendix 4**

Illustrative Indicator Titles by Themes

Note: ^*^Throughout, D-ID refers to dashboard identifiers in Multimedia Appendix 3.

### **I. Public health and epidemiological**

| **Theme** | **Illustrative indicator** | **Example D-ID^*^** |
| --- | --- | --- |
| **Spread and death** | | |
| Cases (all confirmed cases) | All confirmed cases | 1-12, 14, 16-22, 24-47 |
| Active cases | Total active cases | 81, 82, 121-123, 139, 140, |
| Recovered (healed, cured) | Total recovered | 44, 47, 50-53, 87-91 |
| Deaths | Total deaths; number of confirmed deaths | 33-47, 64-99 |
| Mortality rate (case fatality rate) | Lethality rate | 9, 68, 87, 96 |
| Reproduction rates (Rt) (viral reproduction, attack rate) | Instantaneous reproductive num (Rt); Reproduction rate R | 17, 22, 135, 137, 151 |
| Doubling rate | Cases doubling; doubling rate | 3, 12, 17, |
| Future projections/risk models | Scenario projections | 10, 11, 19, 49, 98 |
| **Testing** | | |
| Testing (total number tested, PCR tests) | Total number of tests | 7, 10, 63 |
|  | New tests | 18 |
| Testing rates (positivity, negative tests) | Persons tested positive and negative | 66 |
|  | Number of samples and anlyses carried out, negative and positive cases | 19 |
| Tests-pending results | Suspected case | 138 |
|  | Number of suspected cases assessed by medic in person | 72 |
|  | Number of laboratorial results pending | 93 |
|  | Testing turn around < 24 h | 42 |
| COVID-19 antibody tests (serology tests) | Serology (antibody) surveillance in US | 8 |
| **Risk management** | | |
| Self-quarantine (isolation notices) | Number of people released from isolation | 103 |
|  | Confirmed cases in epidemiological surveillance by health authorities | 93 |
|  | Number of people in quarantine | 88 |
|  | Managed isolation cases | 62 |
|  | Quarantine and managed isolation figures | 63 |
| Contact tracing | Contacts reached within 24 hours | 131 |
|  | COVID-19 check app responses (daily, cumulative) | 123 |

### **II. Health system management**

| **Theme** | **Illustrative indicator** | **Example D-ID** |
| --- | --- | --- |
| **Hospital care** | | |
| Hospitalized (admissions, discharge, under treatment) | Total number of patients released from hospital | 7 |
|  | Total number of hospitalizations | 8, 10, 18, 19, 37 |
|  | Evolution of the number of current COVID-19 related hospitalizations in Quebec by the type of hospital stay | 20 |
|  | Total number of patients under treatment | 26 |
| Admitted to ICU (critical condition) | Number of COVID-19 patients in ICU | 9, 10, 37 |
|  | Proportion of all hospitalizations admitted to the ICU | 39 |
| On a ventilator | Number of patients on a respirator | 34, 42, 66, 79, 100, 102, 127 |
|  | Hospitalizations requiring medical ventilation | 39 |
|  | Current interventions (% and number intubated) | 44 |
|  | Estimated bed/ICU bed/ventilator use (per day) | 98 |
| **Health system capacity** | | |
| Hospital bed capacity (availability) | Percent of hospital beds used | 10 |
|  | General care bed availability | 15, 37, 128 |
|  | Hospital bed occupancy rate | 22, 44, 56, 79, 130 |
|  | Hospital capacity | 51 |
|  | Available hospital beds occupied by confirmed and suspected COVID patients | 94 |
| ICU bed capacity | Percent of ICU beds used (occupied) | 10, 130, 135 |
|  | Intensive care unit (bed with ventilator) availability | 15, 128, 37, 56 |
|  | Total ICU beds occupied; ICU beds left | 42 |
|  | ICU bed occupancy rate | 44 |
|  | Potential/anticipated shortage of ICU beds | 94 |
| Ventilator capacity (available ventilators) | Ventilators available (percentage) | 37 |
|  | Ventilators left | 42, 79 |
|  | ICU-ventilator bed occupancy rate | 44, 130 |
| Personal protective equipment (PPE) and testing stock | Laboratory capacity | 51 |
|  | Equipment distributed (N-95, respirators, procedure masks, gowns, face shields, gloves) in absolute numbers | 37 |
|  | Estimated number of days of PPE available in Toronto hospitals (N95 masks, surgical masks, PPE eyewear, gloves) | 44 |
| Non-COVID service usage | Rate of ER admissions (daily, weekly, men, women)  Rate of hospitalizations  Rate of medical acts; number of COVID related medical acts; number of medical acts for all causes | 23 |
|  | Emergency department visits | 85 |
|  | Accident and emergency waiting times | 108 |

### **III. Social and economic impact**

| **Theme** | **Illustrative indicator (source)** | **Example D-ID** |
| --- | --- | --- |
| Employment and hardship relief | Unemployment rate (monthly) | 78 |
|  | Monthly online job advertisements index (Ministry of Business, Innovation and Employment) | 63 |
|  | Special needs grants for food (Ministry of Social Development) | 63 |
|  | Temporary additional support and special benefit (Ministry of Social Development) | 63 |
|  | Tenants of private single room occupancy hotels or low-income housing receiving daily food support | 118 |
|  | Weekly job postings by sector (Burning Glass Data) | 42 |
|  | Top ten aid from solidarity fund broken down by classification of economic activities (in million €) (Inter-ministerial Digital Directorate) | 22 |
|  | Childcare for essential workers (cumulative) | 118 |
| Transport, trade and international travel | Customs daily border crossing - arrivals (New Zealand Customs Service) | 63 |
|  | Total number of people on student visa; work visa (Ministry of Business, Innovation and Employment) | 63 |
|  | Manufacturing shipments (monthly) (Statistics Canada/Haver Analytics) | 78 |

###

### **IV. Behavioral insights**

| **Theme** | **Illustrative indicator (source)** | **Example D-ID** |
| --- | --- | --- |
| Self-reported adherence to restrictions | Prevalence of systematic adoption of wearing a mask in public during the COVID-19 epidemic (%; weighted data) (CoviPrev survey) | 23 |
|  | In the past 7 days, how often did you practice physical distance with individuals outside your social circle (EKOS Polling) | 130 |
|  | In the past 7 days, how often did you gather with individuals outside your social circle (EKOS Polling) | 130 |
|  | In the past 7 days, how often did you use a mask in indoor public places (EKOS Polling) | 130 |
| Observed public adherence to restrictions | 7-day average percentage change in routing (direction) requests since January 13, 2020 by driving, transit, walking (Apple Mobility) | 42 |
|  | Percentage change in number of visits to various locations (grocery and pharmacy, parks, residential, retail and recreational, transit stations, workplace) in Ontario compared to baseline value (Google Mobility) | 42 |
|  | COVID-19 property-use complaints received by 3-1-1 (hotline) (total) | 118 |
|  | Warnings issued about physical distancing in parks and beaches (total) | 118 |
|  | Vehicle traffic in and out of Vancouver compared to same week in 2019 | 118 |
|  | Bicycle traffic at key locations compared to same week in 2019 | 118 |
|  | Pedestrian traffic at key locations compared to same week in 2019 | 118 |
| Self-reported health and well-being status | Respondents who said their overall wellbeing at the current alert level is worse than usual (New Zealand Health Survey) | 63 |
|  | Percent of respondents who reported experiencing a COVID-19 related scam, over the past 7 days (New Zealand Health Survey) | 63 |
|  | Respondents’ ability to meet bills and other financial commitments over next 3 months (New Zealand Health Survey) | 63 |
|  | Respondents who felt lonely or isolated at least a little of the time over the past 7 days (New Zealand Health Survey) | 63 |
|  | Respondents who said they are either somewhat or completely satisfied with life these days (New Zealand Health Survey) | 63 |
