# Supplementary material for: Features Constituting Actionable COVID-19 Dashboards: Descriptive Assessment and Expert Appraisal of 158 Public Web-Based COVID-19 Dashboards
Source: J Med Internet Res. 2021 Feb 24;23(2):e25682. doi: 10.2196/25682 (PMC7906125; doi:10.2196/25682)
Supplement: Multimedia Appendix 5 [file jmir_v23i2e25682_app5.docx]

**Multimedia Appendix 5**

Summary of Dashboard Scoring

**Table A5.1.** Distribution of scoring across the panel of scorers

|  | Mean score lower |  | Mean score the same |  | Mean score increased |
| --- | --- | --- | --- | --- | --- |

| **Panel of scorers** | **# of dashboards reviewed** | **Mean score^a^ round one** | **Mean score^a^**  **round two** |
| --- | --- | --- | --- |
| 1 | 10 | 4.30 | 4.10 |
| 2 | 10 | 3.90 | 3.80 |
| 3 | 5 | 3.80 | 3.70 |
| 4 | 9 | 3.67 | 3.60 |
| 5 | 10 | 3.50 | 3.44 |
| 6 | 12 | 3.42 | 3.30 |
| 7 | 8 | 3.25 | 3.25 |
| 8 | 10 | 3.10 | 3.08 |
| 9 | 10 | 3.00 | 2.90 |
| 10 | 8 | 2.75 | 2.78 |
| 11 | 7 | 2.71 | 2.75 |
| 12 | 10 | 2.70 | 2.70 |
| 13 | 9 | 2.67 | 2.60 |
| 14 | 10 | 2.60 | 2.60 |
| 15 | 10 | 2.40 | 2.57 |
| 16 | 9 | 2.22 | 2.44 |
| 17 | 11 | 2.09 | 1.81 |
| **Total** | **158** | **3.09** | **3.02** |

^a^The range in mean scores (1.81, 4.10) may be accounted for in part by the sample of dashboards assigned to each panelist. This distribution was determined by the language competencies of panelists and therefore, the set of dashboards reviewed typically reflected a specific sub-set of countries or region.

**Fig. A5.1.** Distribution of scores in round one and two of scoring
